# Psychosis Polyrisk Score and Polygenic Risk Score to Improve Detection and Prognosis in Individuals at Clinical High Risk for Psychosis: Model Development and Internal Validation

**DOI:** 10.1093/schbul/sbag088

**Published:** 2026-08-24

**Authors:** Dominic Oliver, Maite Arribas, Yanakan Logeswaran, Laura Fusar-Poli, Matthew J Kempton, Emily P Hedges, Clara Albinaña, Evangelos Vassos, Amir Sariaslan, William Pettersson-Yeo, Lucia Valmaggia, Mark van der Gaag, Lieuwe de Haan, Barnaby Nelson, Patrick McGorry, Anita Riecher-Rössler, Erich Studerus, Rodrigo Bressan, Neus Barrantes-Vidal, Marie-Odile Krebs, Merete Nordentoft, Stephan Ruhrmann, Gabriele Sachs, Bart Rutten, Jim van Os, Daniel Stahl, Philip McGuire, Paolo Fusar-Poli, for the EU-GEI High Risk Study Group

**Affiliations:** Department of Psychiatry, University of Oxford, Oxford OX3 7JX, United Kingdom; NIHR Oxford Health Biomedical Research Centre, Oxford OX3 7JX, United Kingdom; OPEN Early Detection Service, Oxford Health NHS Foundation Trust, Oxford OX3 7JX, United Kingdom; Early Psychosis: Interventions and Clinical-Detection (EPIC) Lab, Department of Psychosis Studies, Institute of Psychiatry, Psychology & Neuroscience, King's College London, London SE5 8AF, United Kingdom; Early Psychosis: Interventions and Clinical-Detection (EPIC) Lab, Department of Psychosis Studies, Institute of Psychiatry, Psychology & Neuroscience, King's College London, London SE5 8AF, United Kingdom; Early Psychosis: Interventions and Clinical-Detection (EPIC) Lab, Department of Psychosis Studies, Institute of Psychiatry, Psychology & Neuroscience, King's College London, London SE5 8AF, United Kingdom; Department of Biostatistics and Health Informatics, Institute of Psychiatry, Psychology & Neuroscience, King’s College London, London SE5 8AF, United Kingdom; Department of Brain and Behavioral Sciences, University of Pavia, Pavia 27100, Italy; Department of Psychosis Studies, Institute of Psychiatry, Psychology and Neuroscience, King’s College London, London SE5 8AF, United Kingdom; Department of Psychosis Studies, Institute of Psychiatry, Psychology and Neuroscience, King’s College London, London SE5 8AF, United Kingdom; Oxford Big Data Institute, Li Ka Shing Centre for Health Information and Discovery, University of Oxford, Oxford OX3 7LF, United Kingdom; Social, Genetic and Developmental Psychiatry Centre, Institute of Psychiatry, Psychology and Neuroscience, King's College London, London SE5 8AF, United Kingdom; Department of Psychiatry, University of Oxford, Oxford OX3 7JX, United Kingdom; Department of Psychiatry, University of Oxford, Oxford OX3 7JX, United Kingdom; Department of Psychology, Institute of Psychiatry, Psychology & Neuroscience, King's College London, London SE5 8AF, United Kingdom; Centre for Youth Mental Health, University of Melbourne, Parkville, VIC 485 3052, Australia; Orygen, Parkville, VIC 3052, Australia; Faculty of Behavioural and Movement Sciences, Department of Clinical Psychology and EMGO+ Institute for Health and Care Research, VU University, 1081 BT Amsterdam, The Netherlands; Department of Psychosis Research, Parnassia Psychiatric Institute, 2512 HN The Hague, The Netherlands; Department Early Psychosis, Amsterdam UMC, 1105 AZ Amsterdam, The Netherlands; Arkin, 1033 NN Amsterdam, The Netherlands; Centre for Youth Mental Health, University of Melbourne, Parkville, VIC 485 3052, Australia; Orygen, Parkville, VIC 3052, Australia; Centre for Youth Mental Health, University of Melbourne, Parkville, VIC 485 3052, Australia; Orygen, Parkville, VIC 3052, Australia; Faculty of Medicine, University of Basel, CH-4056 Basel, Switzerland; Institute for Information Systems, University of Applied Sciences and Arts Northwestern Switzerland, CH-5210 Basel, Switzerland; LiNC - Lab Interdisciplinar Neurociências Clínicas, Depto Psiquiatria, Escola Paulista de Medicina, Universidade Federal de São Paulo – UNIFESP, São Paulo - SP, 04023-062, Brazil; Departament de Psicologia Clínica i de la Salut (Universitat Autònoma de Barcelona), Centro de Investigación Biomédica en Red de Salud Mental, Instituto de Salud Carlos III (CIBERSAM), Barcelona 08193, Spain; University Paris Cité, GHU Paris, C’JAAD, Inserm UMR1266, Institut de Psychiatrie (CNRS 3557), 75014 Paris, France; Copenhagen Research Center for Mental Health, Mental Health Center Copenhagen, Mental Health Services in the Capital Region of Copenhagen, Copenhagen University Hospital, Copenhagen 2100, Denmark; Department of Psychiatry and Psychotherapy, Faculty of Medicine and University Hospital, University of Cologne, 50931 Cologne, Germany; Department of Psychiatry and Psychotherapy, Medical University of Vienna, 1090 Vienna, Austria; Department of Psychiatry and Neuropsychology, School for Mental Health and Neuroscience, Maastricht University Medical Centre, 6200 MD 464 Maastricht, The Netherlands; Department of Psychiatry and Neuropsychology, School for Mental Health and Neuroscience, Maastricht University Medical Centre, 6200 MD 464 Maastricht, The Netherlands; Department of Biostatistics and Health Informatics, Institute of Psychiatry, Psychology & Neuroscience, King’s College London, London SE5 8AF, United Kingdom; Department of Psychiatry, University of Oxford, Oxford OX3 7JX, United Kingdom; NIHR Oxford Health Biomedical Research Centre, Oxford OX3 7JX, United Kingdom; OPEN Early Detection Service, Oxford Health NHS Foundation Trust, Oxford OX3 7JX, United Kingdom; Early Psychosis: Interventions and Clinical-Detection (EPIC) Lab, Department of Psychosis Studies, Institute of Psychiatry, Psychology & Neuroscience, King's College London, London SE5 8AF, United Kingdom; Department of Biostatistics and Health Informatics, Institute of Psychiatry, Psychology & Neuroscience, King’s College London, London SE5 8AF, United Kingdom; South London and the Maudsley NHS National Health Service Foundation Trust, London SE11 5DL, United Kingdom; Department of Psychiatry and Psychotherapy, Ludwig-Maximilian University, 80336 Munich, Germany

**Keywords:** psychosis, prevention, risk factors, detection, prognosis, environment, genetics

## Abstract

**Background and Hypothesis:**

Psychosis prevention can be supported by tools that facilitate the early detection of individuals at clinical high risk (CHR-P) and predicting their clinical outcomes. We aimed to use clinical, genetic, and environmental data to: (1) predict case–control status as a proof-of-concept for CHR-P detection, and (2) predict transition to psychosis.

**Study Design:**

We used data from the European Network of National Schizophrenia Networks Studying Gene–Environment Interactions: a multicenter cohort study comprising 344 CHR-P individuals and 67 healthy controls. To predict CHR-P status, we used environmental (Psychosis Polyrisk Score [PPS]) and genetic (polygenic risk score for schizophrenia [PRS]) measures with logistic regression (LR) and random forest (RF). To predict transition to psychosis, we used clinical, environmental, and genetic measures with Cox proportional hazards model and random survival forest. Primary outcomes were discrimination (C-index) and calibration (intercept and slope) in repeated nested cross-validation. Clinical utility was assessed with decision curve analysis.

**Study Results:**

For detection of CHR-P, both PPS (LR:C = 0.91, 95% CI, 0.87-0.94, intercept = 1.46, slope = 0.73; RF:C = 0.77, 95% CI, 0.61-0.90, intercept = −0.53, slope = 0.42) and PPS + PRS (LR:C = 0.89, 95% CI, 0.85-0.92, intercept = 1.45, slope = 0.73; RF:C = 0.78, 95% CI, 0.65-0.89, intercept = −0.24, slope = 0.44) had excellent discrimination performance, whereas PRS performed substantially worse (LR:C = 0.60, 95% CI, 0.52-0.67, intercept = 1.63, slope = 0.81; RF:C = 0.57, 95% CI, 0.41-0.72, intercept = −0.84, slope = 0.10). All models over-estimated risk in individuals with low observed risk. Prognosis model performance was poor (C ≤ 0.65).

**Conclusions:**

CHR-P detection may be improved by using the PPS, though evidence is needed from more representative detection settings. However, we did not find evidence that baseline clinical, environmental and/or genetic data enhanced the prediction of psychosis onset.

## Introduction

Psychosis can have a devastating impact on the affected individuals, their families, and communities.^1-3^ Early detection through the clinical high risk for psychosis (CHR-P) state is the most promising strategy to delay or prevent the onset of a first episode of psychosis (FEP).^4^^,^^5^ The effectiveness of the CHR-P state is dependent on efficient detection of CHR-P individuals, differentiating them from healthy peers, and accurate prognosis of psychosis onset.^6^ However, CHR-P services only currently identify 5%-13% of those who develop FEP^7^ and, while the prognostic accuracy of CHR-P assessments is excellent at the population level,^8^ many CHR-P individuals do not develop psychosis.^9^

There is growing evidence that environmental factors, such as childhood trauma, urbanicity, and cannabis use, directly exacerbate neurobiological abnormalities to cause psychosis onset,^10^ and accumulate through the course of psychosis.^10-12^ Some approaches to capturing environmental psychosis risk have been tested, such as the Maudsley environmental risk score^13^ and the exposome score for schizophrenia,^14^ but have been restricted to a small number of non-systematically selected risk factors. The exposome shows good discrimination between patients with psychosis and healthy controls^15-17^ as well as being a marker of poor functioning in psychosis.^18^ The Psychosis Polyrisk Score (PPS) was conceptualized as an instrument that evaluates environmental^19^ risk factors for psychosis at a multivariate level, systematically identified from umbrella reviews and meta-analyses.^12^^,^^19^ In pilot analyses using meta-analytic weightings, individuals referred for a clinical CHR-P assessment had higher PPS scores compared to healthy controls.^20^ The PPS concept has not yet been applied to formally develop or validate a clinical prediction model in a CHR-P population.

The polygenic risk score for schizophrenia (PRS) similarly indexes psychosis risk associated with the cumulative effects of single nucleotide polymorphisms across the genome.^21^ Previous research has found that the PRS alone only explains 9.4% of variance between patients with FEP and healthy controls^22^ and only 3.5%-12.3% of transition risk in CHR-P individuals (*R*^2^_liability_).^23^ Given that psychosis risk is likely to be a function of interactions and correlations between genetic and the environmental factors,^24-26^ assessing interactions between PRS and PPS may provide a more comprehensive measure of psychosis risk than using either type of data alone.

In this study, we aimed to combine clinical, PRS, and PPS data for the first time to develop and validate clinical prediction models to discriminate between CHR-P individuals and healthy controls as a proxy for detection that maximizes statistical sensitivity as a proof of concept, and to predict transition to psychosis.

## Methods

### Participants

Participants were recruited to an international multicenter prospective cohort study (European Network of Schizophrenia Networks for the Study of Gene Environment Interactions; EU-GEI)^27^ between May 2010 and April 2015 (Methods S1).

### Predictors

We included 3 predictor sets: clinical, environmental (PPS), and genetic (PRS).

Three detection models were fitted: (1) PPS predictors only; (2) PRS only; (3) PPS predictors and PRS combined. Seven prognosis models were fitted: (1) clinical predictors only; (2) PPS predictors only; (3) PRS only; (4) clinical and PPS predictors; (5) clinical predictors and PRS; (6) PPS predictors and PRS; (7) clinical, PPS, and PRS combined.

### Clinical

Three clinical predictors were included in prognosis models: (1) baseline CAARMS positive symptom scores and Global Assessment of Functioning (GAF), (2) symptoms, and (3) disability subscales.

### P‌PS

Due to some environmental risk factors incorporating a combination of genetic and environmental components, a distinction between the 2 may be spurious. We therefore define environmental risk factors as non-purely-genetic risk factors, which can be categorized as sociodemographic, parental, perinatal, later factors, and antecedents.

Overall, 21 non-purely-genetic predictors were included from the original PPS concept,^20^ selected on the basis of the strongest available evidence for their association with psychosis onset.^19^ Details about the systematic PPS factor selection and operationalization are presented in Methods S2 and S3 and Table S1.

These were derived from 11 standalone factors that had available data: (1) male gender and aged 25-35; (2) non-right-handedness; (3) low paternal socioeconomic status; (4) parental severe mental illness; (5) adult life events; (6) heavy cannabis use; (7) childhood trauma; (8) trait anhedonia; (9) urbanicity; (10) pollution; (11) tobacco use.

These were supplemented by 6 predictors indexing ethnicity and ethnic density: (12) White ethnicity; (13) Black ethnicity and low ethnic density; (14) Black ethnicity and high ethnic density; (15) Other ethnicity and low ethnic density; (16) Other ethnicity and medium ethnic density; (17) Other ethnicity and high ethnic density.

These were supplemented by 4 predictors indexing immigration: (18) first generation immigrant from North Africa; (19) first generation immigrant other; (20) second generation immigrant from North Africa; (21) second generation immigrant other.

### PRS

Blood samples were collected from all participants at baseline and genotyped as previously described.^22^ After quality control, we imputed genetic variants for 383 individuals with genotype information in EU-GEI (Methods S3). The PRS for schizophrenia was built by SBayesRC^28^ software using the latest gene wide association study (GWAS) summary statistics from the Psychiatric Genomics Consortium on schizophrenia.^29^ We performed principal component analysis on the genetic data and included the first 10 principal components (PCs) across models to control for the effects of population stratification.

### Outcomes

For detection models, the outcome was meeting CHR-P criteria at baseline as defined by the CAARMS.^30^

For prognosis models, the outcome was transition to psychosis over the course of follow-up, defined either psychometrically by CAARMS criteria^30^ or clinically by ICD-10^31^ criteria, DSM-IV^32^ criteria or accepted referral to an early intervention in psychosis clinical team ascertained by contact with the clinical team and/or from review of electronic health records.

### Statistical Analysis

Model development and internal validation followed the Steyerberg et al guidelines^33^ and the Transparent Reporting of a Multivariable Prediction Model for Individual Prognosis or Diagnosis (TRIPOD+AI; Table S2).^34^ All statistical analyses were performed using R version 4.2.2. All analysis code is publicly available on GitHub: https://github.com/dapoliver/PPS_EUGEI.

We first performed descriptive analyses of baseline clinical and sociodemographic characteristics of the sample, obtaining means and frequencies for continuous and categorical variables, respectively. The cumulative incidence of psychosis was visualized with the Kaplan–Meier failure function (1-survival)^35^ and Greenwood 95% CIs,^36^ conducted using the survival (version 3.5-7) and survminer (version 0.4.9) packages. Follow-up was censored at 3 years.

### Model Development

Detection models were developed using both healthy control and CHR-P data, whereas prognosis models solely used CHR-P data. No predictors were transformed. The detection models were fitted using (1) logistic regression with Least Absolute Shrinkage and Selection Operator (LASSO; to shrink coefficients of redundant predictors to zero) (glmnet [version 4.1-6]; DMwR2 [version 0.0.2]); (2) random forest (caret [version 6.0-93]) and the prognosis models using (1) Cox proportional hazards models with LASSO (glmnet); (2) random survival forests (randomforestSRC [version 3.2.0]). All models initially outputted probabilities, with a threshold of 0.5 used for classification measures (eg, sensitivity). Sample size calculations^37^^,^^38^ were performed post hoc to use accurate parameters.

We initially fitted models on all available data to investigate (1) the proportion of variance explained by these predictor sets, indexed by Nagelkerke *R*^2^^39^ and *R*^2^_liability_^40^; (2) univariate associations between each predictor and relevant outcomes using logistic regression and Cox proportional hazards models.

### Internal Validation

Internal validation was performed using repeated nested cross-validation, as implemented in caret (version 6.0-93), with 5 inner folds, 5 outer folds, 10 inner repetitions, and 10 outer repetitions. This framework randomly splits the data into 5 outer folds, with 4-folds being used for model training and the remaining fold being used for model testing. The 4 training folds are split into 5 inner folds, where 4 folds are used to tune hyperparameters (features that affect how the model uses the data, for example, lambda values in LASSO dictates how many predictors are dropped from the model) and tested on the remaining test fold so that overfitting is mitigated against while ensuring good performance. This is repeated 10 times for robustness. A final model is fitted on all test data using the best-performing hyperparameters, which is then evaluated on the test data. This process is repeated until all 5-folds have been used as test data. This process is repeated 10 times, generating different folds in each repeat. Due to some sites reporting zero events, we opted against a leave-one-site-out cross-validation framework, where test sets are defined by geographic location rather than randomly selected. Missing data were imputed within the cross-validation framework (Methods S5). Predictors with near zero variance were removed.

### Model Performance

Model performance was primarily assessed through discrimination (Harrell’s C statistic [0.9-1.0 is considered outstanding, 0.8-0.9 excellent and 0.7-0.8 acceptable^41^]) and calibration (calibration intercept [ideally close to 0] and slope [ideally close to 1]). Discrimination is the ability of the model to distinguish between cases and controls, with Harrell’s C indexing the proportion of pairs of randomly selected cases and controls where the case has a higher risk score than the control. Calibration assesses the relationship between predicted probabilities and observed risk proportions (eg, in a sample where 2 out of 10 individuals have the outcome, the average risk estimate should be 20%). This is captured by the calibration intercept, measuring whether a model systematically over- or under-predicts the outcome on average (ideally close to 0) and calibration slope, measuring how well risk estimates map to observed outcomes across different levels of prevalence (ideally close to 1).

We also recorded the Brier score, which is an overall measure of algorithm performance, (ideally close to 0, with scores >0.25 generally indicating a poor model) and integrated calibration index, another calibration metric representing the weighted average between observed and predicted probabilities (ideally close to 0).^42^ We also calculated classification metrics for detection models, including balanced accuracy, sensitivity, specificity, positive predictive value (PPV), and negative predictive value (NPV). Participant-level risk estimates were aggregated to calculate means and 95%CIs for all performance metrics using 2000 bootstraps.

Decision curve analysis^43^ was used to assess the clinical usefulness of models with adequate performance (C ≥ 0.60) by estimating net benefit (Methods S5). The decision curve analysis quantifies the clinical “net benefit” for a clinical prediction model over a range of hypothetical clinical preferences. Net benefit indexes the difference between the clinical benefit of accurate predictions (eg, detecting CHR-P individuals) and the clinical harm of inaccurate predictions (eg, provision of unnecessary preventive care). As different clinical scenarios may correspond to different preferences regarding missing psychosis cases and providing unnecessary interventions, we plotted net benefit across a range of threshold probabilities (0%-50%). Values closer to 0% represent greater concern for missing potential CHR-P individuals and values closer to 50% represent greater concern for providing unnecessary preventive care. The net benefits of using the model were plotted compared to default strategies of assessing all or no individuals at risk.^44^ Standardized net benefit was calculated as the net benefit divided by the incidence of the outcome, showing the percentage increase in net benefit at a given risk threshold. As prevalence of CHR-P individuals is dependent on the underlying population they are sampled from, we tested the net benefit using the prevalence of CHR-P individuals in the general population (1.7%) and in clinical samples (19.2%).^45^

Model coefficients for logistic regression and Cox proportional hazard models were presented after refitting models on all available data to facilitate future external validation. As model coefficients cannot be generated for random forest models, we presented variable importance (VImp) values for these models.

### Sensitivity Analyses

First, due to potential differences in distribution of predictors between sites, we re-ran all models using mean offset to harmonize data across sites.^46^ Second, as the PRS for schizophrenia was derived from a GWAS based on White European-ancestry samples and prediction accuracy decreases with genetic distance to the discovery population,^47^ which can exacerbate health disparities if using PRS in clinical settings,^48^^,^^49^ we re-ran models on individuals with White European ancestry only. Finally, additional prognosis models were run with logistic regression and random forest to predict remission from the CHR-P state at 2 years defined using CAARMS criteria^30^ using the same modelling pipeline.

## Results

Three hundred forty-four CHR-P participants and 67 healthy controls were included (Table 1). The mean follow-up time in the CHR-P cohort was 644.4 (SD = 332.1) days. 223 (64.8%) CHR-P participants and 11 (16.4%) healthy controls met criteria for depression with 176 (51.1%) CHR-P participants and 4 (6.0%) healthy controls meeting criteria for anxiety disorders. During 3 years of follow up, 65 (19.0%) CHR-P individuals had developed a FEP (44 defined psychometrically; 21 defined clinically; Figure S2).

**Table 1:** Sociodemographics

**Predictor**	**Healthy controls (*n* = 67)**	**CHR-P (*n* = 344)**
Age	22.9 (4.1)	22.4 (4.9)
Gender (%male)	34 (50.7%)	185 (53.8%)
Non-right-handedness	4 (9.8%)	20 (10.8%)
*Missing*	*26 (38.8%)*	*159 (46.3%)*
High urbanicity	66 (98.5%)	329 (95.9%)
*Missing*	*2 (3.0%)*	*26 (7.6%)*
High pollution	67 (100.0%)	309 (90.1%)
*Missing*	*20 (29.9%)*	*61 (17.8%)*
**Ethnicity**		
White	42 (62.7%)	247 (71.8%)
Black	10 (14.9%)	34 (9.9%)
Other	15 (22.4%)	63 (18.3%)
**Ethnic density**		
High	55 (84.6%)	130 (50.2%)
Low	9 (13.8%)	115 (44.4%)
*Missing*	*2 (3.0%)*	*85 (24.7%)*
**Immigration**		
First generation immigrant	19 (28.4%)	84 (24.4%)
First generation immigrant from North Africa	0 (0%)	1 (0.3%)
Second generation immigrant	17 (25.4%)	90 (26.2%)
Second generation immigrant from North Africa	0 (0%)	5 (1.5%)
*Missing*	*2 (3.0%)*	*19 (5.5%)*
Paternal age < 35	67 (100.0%)	343 (100.0%)
Low paternal socioeconomic status	8 (12.1%)	63 (19.1%)
*Missing*	*1 (1.5%)*	*14 (4.1%)*
Parental severe mental illness	14 (27.5%)	174 (64.2%)
*Missing*	*16 (23.9%)*	*73 (21.2%)*
Stressful life events	46 (68.7%)	279 (85.8%)
*Missing*	*0 (0%)*	*19 (5.5%)*
Daily tobacco use	17 (25.4%)	175 (51.0%)
*Missing*	*0 (0%)*	*23 (6.7%)*
Heavy cannabis use	9 (13.4%)	111 (33.2%)
*Missing*	*0 (0%)*	*10 (2.9%)*
Childhood trauma	2 (3.0%)	84 (26.3%)
*Missing*	*1 (1.5%)*	*25 (7.3%)*
Trait anhedonia	8 (12.1%)	210 (62.3%)
*Missing*	*1 (1.5%)*	*7 (2.0%)*
PRS	0.00 (1.0)	0.00 (0.9)
*Missing*	*2 (3.0%)*	*24 (7.0%)*
CAARMS-P	0.6 (1.3)	9.9 (5.1)
GAF symptoms	85.9 (10.9)	55.1 (10.1)
*Missing*	*1 (1.5%)*	*27 (7.8%)*
GAF disability	84.9 (9.0)	55.4 (12.0)
*Missing*	*1 (1.5%)*	*12 (3.5%)*

Abbreviations: CAARMS-P = Comprehensive Assessment of At Risk Mental States – Positive subscale (severity); CHR-P = clinical high risk for psychosis; GAF = Global Assessment of Functioning; PRS = polygenic risk score for schizophrenia.

Categorical variables are presented as *n* (%) with percentages of non-missing values. Continuous variables are presented as mean (SD).

### Proportion of Variance Explained

#### Variance between Healthy Controls and CHR-P Individuals

**Figure 1:**
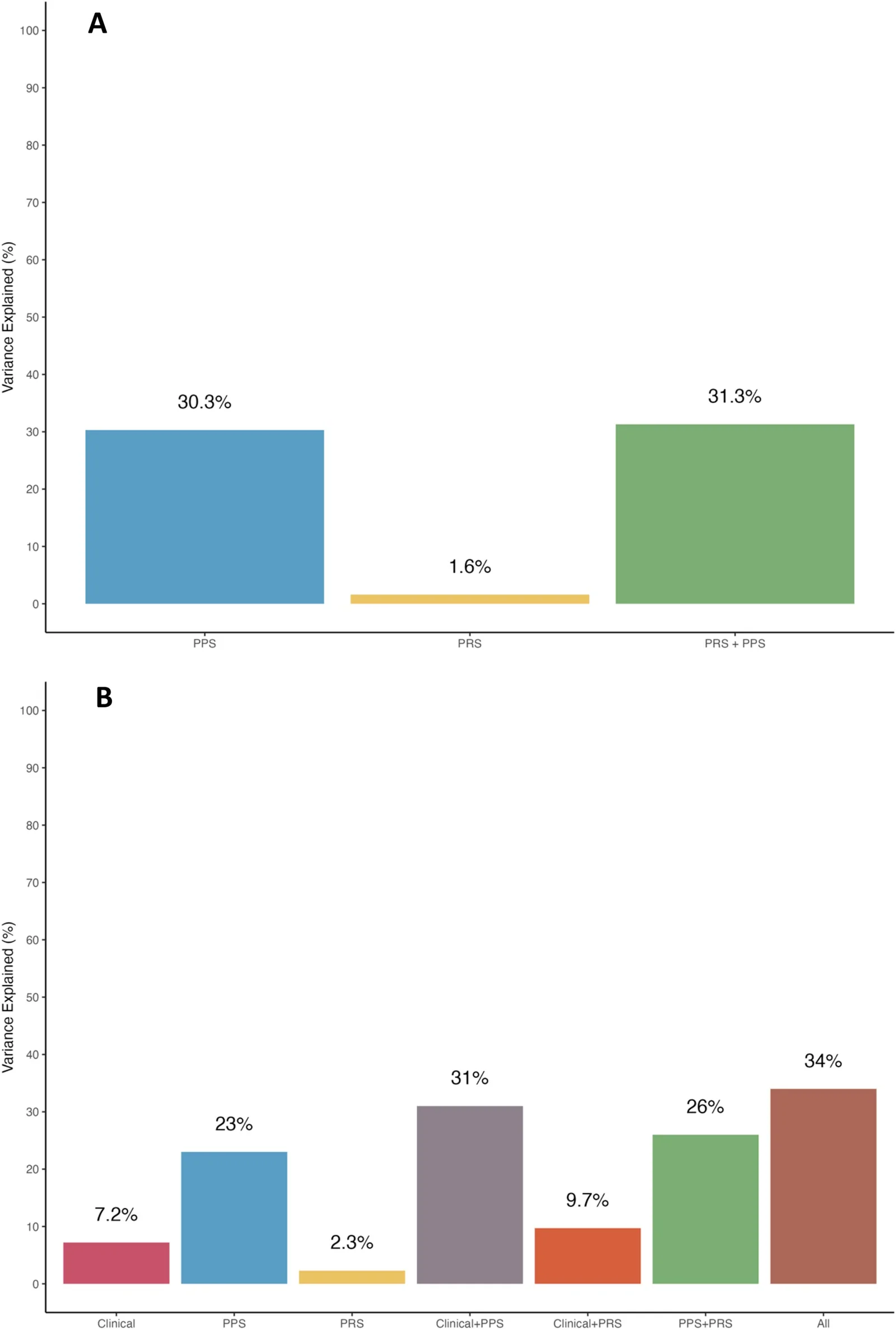
Proportion of Variance in the EU-GEI Sample Explained in (A) Detection models; (B) Prognosis Models.

PRS alone accounted for 1.3% (0.1% *R*^2^_liability_) of the variance between healthy controls and CHR-P individuals, while PPS measures alone accounted for 30.3% (5.0% *R*^2^_liability_). The combination of PRS and PPS measures accounted for 31.3% (5.2% *R*^2^_liability_) of the variance (Figure 1).

#### Variance in Transition Risk in CHR-P Individuals

Clinical data alone accounted for 7.2% (3.6% *R*^2^_liability_) of the variance in transition risk within the CHR-P sample, while PRS alone accounted for 2.3% (1.9% *R*^2^_liability_), and PPS measures alone accounted for 23% (21.8% *R*^2^_liability_). The combination of PRS and PPS accounted for 26.0% (26.3% *R*^2^_liability_) of the variance, compared to 9.7% (5.7% *R*^2^_liability_), for clinical and PRS combined, and 31.0% (28.7% *R*^2^_liability_) for clinical and PPS combined. Combining clinical, PRS, and PPS accounted for 34.0% (34.1% *R*^2^_liability_) (Figure 1).

### Univariate Models

CHR-P individuals were more likely to endorse childhood trauma (OR = 14.5, 95% CI, 2.9-63.4, *P* = .001), trait anhedonia (OR = 12.4, 95% CI, 5.7-26.7, *P* < .001), parental severe mental illness (OR = 5.3, 95% CI, 2.7-9.5, *P* < .001), heavy cannabis use (OR = 3.3, 95% CI, 1.5-9.5, *P* = .002), tobacco use (OR = 3.1, 95% CI, 1.7-5.5, *P* < .001), adult life events (OR = 2.8, 95% CI, 1.5-5.2, *P* = .001) and higher PRS (OR = 2.4, 95% CI, 1.2-4.6, *P* = .01) compared to healthy controls (Table S3).

Most predictors were not significantly associated with transition to psychosis (*P* > .05), with the exception of GAF symptoms, which showed protective effects with higher scores (HR = 0.97, *P* = .04) (Table S4).

### Prediction Modeling

#### Detection

Most PPS predictors were retained across multivariate models, with only non-right-handedness and first-generation immigrants from North Africa dropped by LASSO across PPS and PPS + PRS models. PRS was retained in the PPS + PRS model. Model coefficients are presented in Table S5.

**Figure 2:**
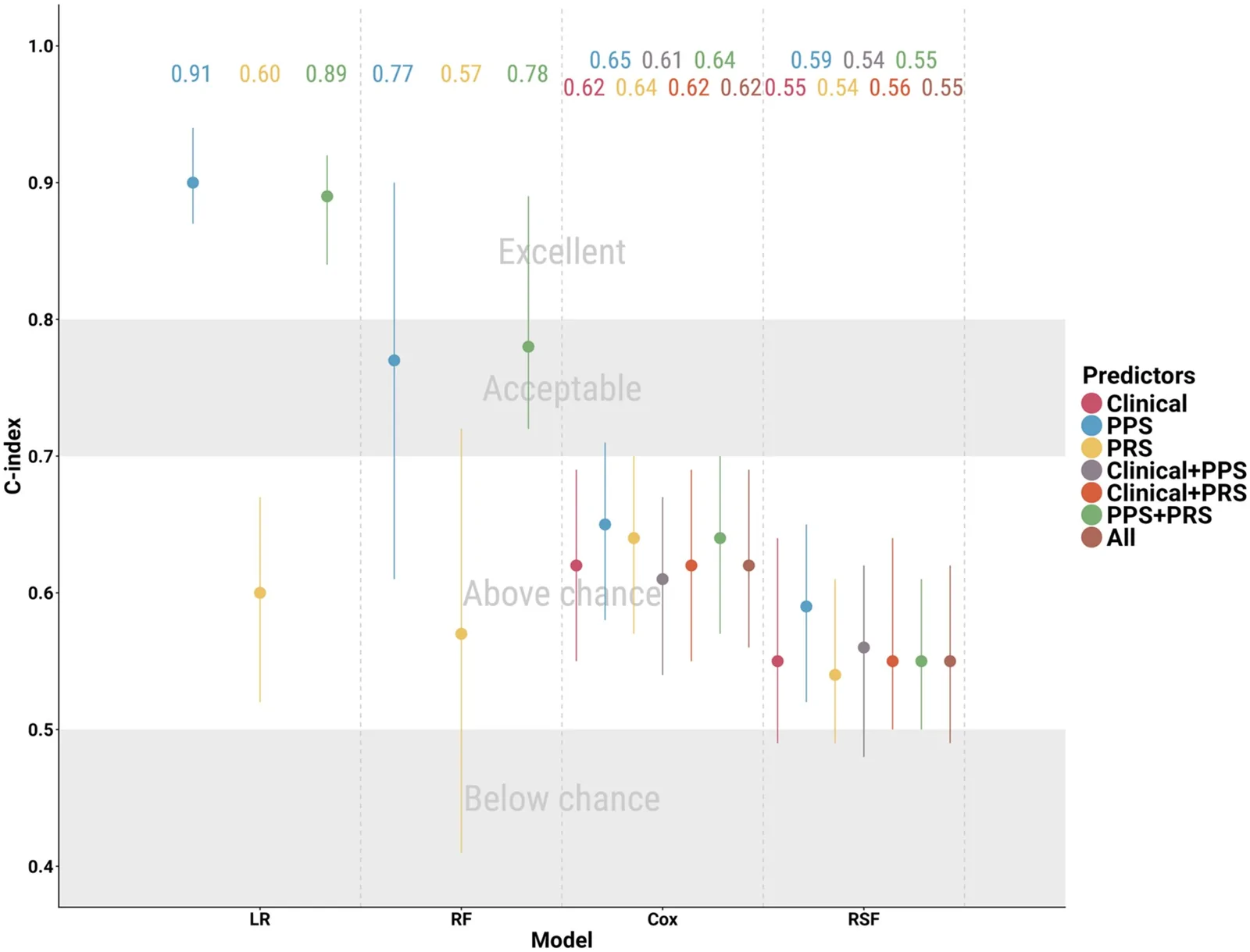
Discrimination Performance (Measured by C-Index) Across Fitted Detection (Logistic Regression [LR] and Random Forest [RF]) and Prognosis (Cox Proportional Hazards Model [Cox] and Random Survival Forest [RSF]).

For logistic regression models, PPS predictors alone (C = 0.91, 95% CI, 0.87-0.94) and PPS + PRS (C = 0.89; 95% CI, 0.85-0.92) performed similarly well in terms of discrimination (Table 2; Figure 2). PRS alone produced significantly lower discrimination performance (C = 0.60, 95% CI, 0.52-0.67) (Table 2; Figure 2).

**Table 2:** Model Performance

**Detection**										
**Model**	**C-index**	**Balanced Accuracy**	**Sensitivity**	**Specificity**	**PPV**	**NPV**	**Calibration Intercept**	**Calibration Slope**	**Brier Score**	**ICI**
**Logistic regression**										
**PPS**	0.905 (0.872-0.935)	82.7% (77.4%-87.5%)	84.9% (80.8%-88.7%)	80.5% (70.1%-89.6%)	95.7% (93.6%-97.6%)	51.1% (44.4%-58.6%)	1.46 (1.16-1.76)	0.73 (0.58-0.92)	0.12 (0.10-0.14)	0.11 (0.09-0.14)
**PRS**	0.596 (0.522-0.670)	54.9% (48.4%-61.3%)	55.8% (50.6%-61.0%)	54.0% (41.8%-65.7%)	86.2% (82.9%-89.5%)	19.2% (15.3%-23.1%)	1.63 (1.60-1.67)	0.81 (-0.01-1.67)	0.24 (0.24-0.25)	0.33 (0.32-0.34)
**PPS + PRS**	0.885 (0.847-0.921)	80.9% (75.4%-86.0%)	82.9% (78.5%-86.6%)	79.0% (68.7%-88.1%)	95.3% (93.1%-97.3%)	47.5% (41.1%-54.5%)	1.45 (1.17-1.72)	0.73 (0.58-0.91)	0.13 (0.10-0.15)	0.12 (0.10-0.15)
**Random forest**										
**PPS**	0.767 (0.605-0.904)	61.7% (50.9%-73.6%)	92.3% (84.8%-98.5%)	31.0% (12.5%-56.2%)	84.7% (80.8%-89.2%)	50.2% (21.4%-81.8%)	−0.53 (−1.11 to 0.06)	0.42 (0.11-0.89)	0.14 (0.10-0.18)	0.13 (0.08-0.18)
**PRS**	0.567 (0.410-0.717)	48.5% (46.2%-50.0%)	96.9% (92.4%-100%)	0.0% (0.0%-0.0%)	80.0% (79.2%-80.5%)	0.0% (0.0%-0.0%)	−0.84 (−1.31 to −0.43)	0.10 (−0.13 to 0.36)	0.18 (0.15-0.20)	0.15 (0.10-0.21)
**PPS + PRS**	0.776 (0.654-0.886)	60.9% (49.5%-73.5%)	90.9% (83.3%-97.0%)	31.0% (12.5%-56.2%)	84.5% (80.3%-89.4%)	45.8% (18.7%-76.9%)	−0.24 (−0.87 to 0.41)	0.44 (0.21-0.77)	0.15 (0.10-0.20)	0.11 (0.05-0.17)
**Prognosis**										
**Model**	**C-index**		**Calibration intercept**		**Calibration slope**		**Brier score**		**ICI**	
**Cox proportional hazards model**										
**Clinical**	0.617 (0.547-0.685)		−0.94 (−0.96 to −0.92)		12.08 (6.40-17.84)		0.17 (0.16-0.17)		0.17 (0.16-0.19)	
**PPS**	0.649 (0.582-0.711)		−1.52 (−1.54 to −1.50)		9.69 (6.20-14.55)		0.23 (0.23-0.24)		0.31 (0.30-0.32)	
**PRS**	0.636 (0.569-0.703)		−1.58 (−1.61 to −1.55)		5.08 (2.66-7.60)		0.24 (0.23-0.24)		0.32 (0.31-0.33)	
**Clinical + PPS**	0.605 (0.539-0.670)		−1.25 (−1.26 to −1.23)		9.47 (3.94-14.90)		0.20 (0.19-0.20)		0.24 (0.23-0.25)	
**Clinical + PRS**	0.619 (0.546-0.687)		−0.93 (−0.95 to −0.91)		12.21 (6.32-18.37)		0.16 (0.16-0.17)		0.17 (0.16-0.18)	
**PPS + PRS**	0.637 (0.572-0.699)		−1.54 (−1.56 to −1.52)		8.71 (5.25-13.00)		0.23 (0.23-0.24)		0.31 (0.30-0.32)	
**All**	0.622 (0.556-0.685)		−1.29 (−1.30 to −1.27)		10.68 (5.53-15.82)		0.20 (0.20-0.20)		0.25 (0.24-0.26)	
**Random survival forest**										
**Clinical**	0.550 (0.494-0.635)		−0.31 (−0.38 to −0.24)		1.67 (−1.24 to 4.66)		0.15 (0.14-0.16)		0.08 (0.05-0.11)	
**PPS**	0.585 (0.520-0.650)		−0.30 (−0.35 to −0.26)		−3.68 (−6.61 to −0.99)		0.15 (0.15-0.16)		0.10 (0.06-0.13)	
**PRS**	0.539 (0.485-0.605)		−0.32 (−0.38 to −0.25)		0.85 (−1.49 to 3.06)		0.15 (0.14-0.16)		0.08 (0.05-0.11)	
**Clinical + PPS**	0.542 (0.483-0.618)		−0.34 (−0.39 to −0.29)		1.12 (−2.22 to 4.75)		0.15 (0.14-0.15)		0.07 (0.05-0.10)	
**Clinical + PRS**	0.562 (0.499-0.640)		−0.40 (−0.46 to −0.33)		1.94 (−0.62 to 4.51)		0.15 (0.14-0.16)		0.08 (0.06-0.11)	
**PPS + PRS**	0.547 (0.495-0.613)		−0.32 (−0.37 to −0.28)		−1.69 (−4.59 to 1.23)		0.15 (0.15-0.16)		0.09 (0.06-0.12)	
**All**	0.546 (0.490-0.618)		−0.36 (−0.40 to −0.31)		1.48 (−1.91 to 4.88)		0.15 (0.14-0.15)		0.07 (0.05-0.10)	

Abbreviations: ICI = integrated calibration index; NPV = negative predictive value; PPS = psychosis polyrisk score; PPV = positive predictive value; PRS = polygenic risk score for schizophrenia.

However, there was evidence of miscalibration, typically over-estimating risk in individuals presenting with low observed risk through a high calibration intercept (Table 2).

For random forest models, the pattern of discrimination performance was similar but lower than the logistic regression models, largely driven by poor sensitivity (<60%). These models had better calibration than logistic regression models but showed similar patterns of miscalibration. Variable importance is presented in Table S6.

Decision curve analysis suggested that at certain predicted probability cutoffs, the PPS alone and combined PPS + PRS logistic regression models resulted in a greater net benefit than the competing extremes of intervening in all patients or in none. These models showed marginal net benefit within a restricted selection of risk thresholds in the general population (0.005-0.017).

**Figure 3:**
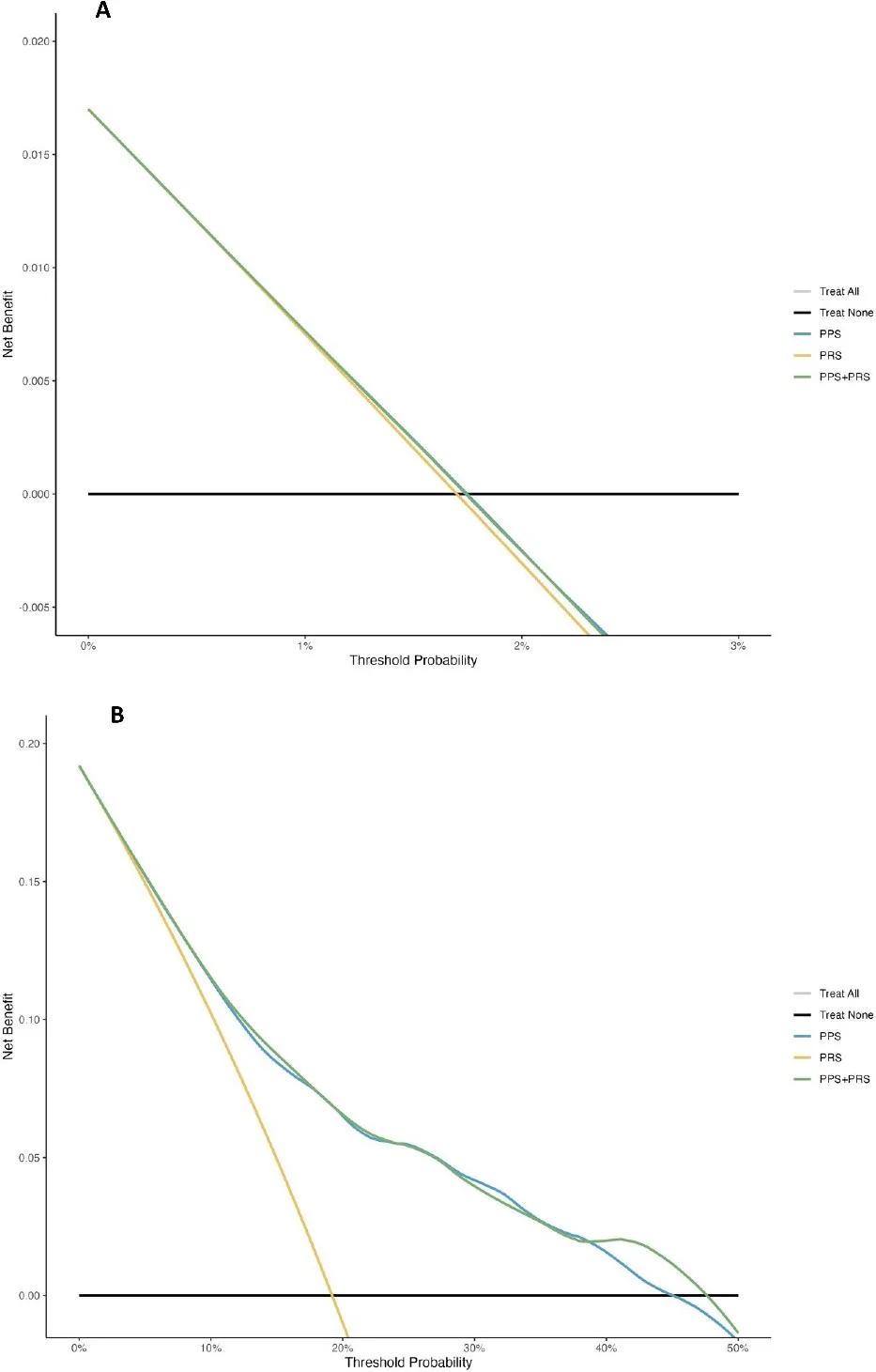
Decision Curve Analysis Plots for Logistic Regression Models. The plot reports net benefit (*y*-axis) of across a range of risk thresholds (*x*-axis) compared with screening all individuals or screening no individuals in (A) the general population and (B) clinical samples.

However, the PPS and PPS + PRS models displayed greater net benefit compared with screening all or screening none between 0 and 0.45 in clinical samples (Table S7; Figure 3). For example, if a CAARMS assessment were considered necessary above a risk score of 0.20 in clinical samples (ie, not detecting a CHR-P individual is 4 times as harmful as an unnecessary assessment), the PPS + PRS would provide a net benefit of 6.6% over screening all, meaning that 33% more CHR-P individuals could be detected. This results in 7 additional CHR-P individuals detected per 100 individuals.

#### Prognosis

Random survival forest showed higher discrimination performance than the Cox proportional hazards models but discrimination was poor (C ≤ 0.65) across all models (Table 2; Figure 2). All models displayed miscalibration, typically under-estimating risk in individuals presenting with high observed risk (Table 2).

Most predictors were dropped by LASSO in the Cox proportional hazards model, and GAF symptom score was the most important predictor in random survival forest models. PPS predictors generally showed low importance in random survival forest models (Tables S8 and S9).

#### Sensitivity Analyses

Models with predictors harmonized across sites and restricted to participants from White European ancestry did not perform significantly differently from the main analyses (Table S10).

Models predicting remission from the CHR-P state performed similarly (Table S11).

Sample size calculations are presented in Results S1. Four out of 10 models were adequately powered (detection: PRS [*n* = 355 required]; prognosis: clinical [*n* = 141 required], PRS [*n* = 445 required] and clinical + PRS [*n* = 205 required]) with the rest having smaller sample sizes than required, including PPS (*n* = 488 required) and PPS + PRS (*n* = 546 required) detection models.

## Discussion

The present study evaluated several models that use the PPS and PRS to improve detection and prognosis of CHR-P individuals. PPS predictors resulted in excellent model performance for discriminating between CHR-P individuals and healthy controls, while the addition of the PRS provided little additional benefit. These results represent a proof-of-concept for the potential of environmental risk factors to aid detection of CHR-P individuals. However, the PPS and PRS did not predict transition to psychosis well, even with the addition of clinical predictor information.

These findings reinforce the potential of the PPS to aid detection of CHR-P individuals.^12^^,^^20^ Current detection strategies for this group only detect a minority (5%-13%) of FEP cases prior to psychosis onset^7^^,^^50^ despite the majority experiencing a detectable prodrome.^51^^,^^52^ The best performing model in this study were the PPS alone with high discrimination (C = 0.88) and clinical utility, with no additional predictive gain with the addition of the PRS. This suggests that the PPS could inform detection of CHR-P individuals similarly well without requiring the ethical, logistic, or financial burden of genetic testing. Pre-screening with digital self-report methods (eg, prodromal questionnaire [PQ-16]) has been shown to be an effective method of increasing risk enrichment when screening in community samples,^53-55^ to the extent that the level of risk is similar to clinically referred samples.^8^ Our results suggest that using the PPS can further enhance the ability of current pre-screening instruments to enrich risk. However, our decision curve analyses indicate that this benefit is limited if applied to the general population and should therefore be implemented in screening samples that already have some enriched risk, such as help-seeking individuals or clinical populations, such as in primary^56^ and secondary care^50^^,^^57-63^ or detected through the PQ-16. The potential benefit of the PPS being incorporated alongside the PQ-16 in participants recruited from the community is currently being investigated through the ENTER study.^64^ This step is essential to understand whether the maximized signal indexed in this study can be applied to improve identification of CHR-P individuals from a pre-screened community sample. The current study artificially introduces a bimodal distribution through its case–control design, meaning that these results may not be reflective of either the general population or help-seeking clinical populations. Moreover, despite high discrimination performance, classification performance, and clinical utility, there was evidence of miscalibration. While these results are promising, they need further replication through external validation in other large, representative samples.^65-67^

In contrast, when used to facilitate prediction of transition to psychosis, the performance of the PPS was poor (C ≤ 0.62) and displayed severe miscalibration. This suggests that the PPS may not be strongly informative for prognosis. This may be due to the fact that the CHR-P population is already enriched for environmental risk factors compared to the general population, as shown in the present results and previous meta-analytic evidence.^11^ For example, the prevalence of childhood trauma increases from 2% in healthy controls to 26% in CHR-P individuals, but is similar in those who developed psychosis (25%) to those who did not (26.6%). As a result, the effect size for differences between CHR-P individuals who transition and those who do not may be smaller, which is more challenging to model. Moreover, exposure to these risk factors is not static,^68^ meaning that the timeline of exposure prior to and following baseline might be integral to accurately modelling risk. These issues ultimately increase the difficulty of identifying the meaningful signals that can inform prognosis in this population. This challenge is likely exacerbated by the limitations of the dataset we examined, which had a relatively low event rate and included participants from a diverse set of sites worldwide (however, harmonization across sites did not impact model performance). Ideally, data from large scale studies from geographically similar sites (eg, PRESCIENT or ProNET^67^) would be better powered to test this. However, this may present an issue with generalizability, with some risk factors solely being informative in certain populations (eg, ethnicity likely has different associated risk outside of countries with a majority White population), which may suggest the utility of more site-specific versions of the PPS, as seen in the Korean Polyenvironmental Risk Score for Psychosis.^69^

While we are not able to accurately predict psychosis onset within CHR-P individuals, detecting individuals at risk still has clinical importance. CHR-P individuals are typically help-seeking for distressing experiences that are impacting daily functioning. Identifying more of these individuals during this vulnerable period enables treatment of presenting symptoms as well as preventive interventions that can alter the course of the disorder. Moreover, currently, the CHR-P population represents a minority of the overall future FEP population.^7^^,^^50-52^ Expanding detection efforts can increase the proportion of patients with FEP detected early. These patients can be referred to early intervention services earlier, reducing duration of untreated psychosis and improving outcomes.^5^^,^^70^ Moreover, expanding samples without diluting risk can facilitate well-powered research into prognostic markers for disorder onset as well as putative preventive therapeutics.

The present study had some limitations. First, considering the relatively large number of predictors in some models (up to 19 in detection models and up to 22 in prognosis models), the sample size is low in some scenarios (*n* = 355-546 needed for detection models; *n* = 360-577 for prognosis).^37^^,^^38^ However, we have mitigated against this by using regularization (LASSO) in logistic regression and Cox proportional hazards models to drop uninformative predictors from models and by using relatively high minimum node sizes and low values of mtry in random forest models. Second, for detection models, the healthy control sample was recruited separately from the CHR-P sample at a subset of sites so we may be modelling sampling biases. Our study was designed to maximize model performance in a case–control sample, which is an important model development step as a proof-of-concept, even though it does not correspond to expected real-world use cases. As a result, it is unclear to what extent the model performance is driven by selection biases between the 2 groups rather than psychosis risk. We have attempted to mitigate this by selecting predictors on the basis of meta-analytic evidence of risk factors for psychosis,^19^ rather than selecting solely on the basis of group differences within the EU-GEI cohort. Relatedly, this means that while our estimates of the proportion of variance explained are representative of our sample, they may not be representative of the underlying population. Replication of these findings in more representative general population or help-seeking clinical samples are essential. Third, some variables (eg, non-right-handedness and cannabis use) had a relatively high proportion of missingness but we found no pattern of missingness and used imputation to maximize utility of the dataset, which would also be needed for prospective use. Fourth, our sample was smaller than required to support development of a robust model. This also inhibited our ability to assess model performance across subgroups (eg, ethnic minority groups), which is particularly relevant for PRS analyses. Despite this, the signal identified in detection models remains promising, particularly given our robust internal validation through repeated nested cross-validation, which increases likelihood of generalizability compared to other internal validation techniques like bootstrapping or k-fold cross-validation.^71^ In addition, we have followed gold standard methodological guidelines for model development.^33^^,^^34^ Despite this, it is crucial to externally validate these findings in independent datasets,^65^^,^^72^^,^^73^ and potentially refit the models in larger samples (eg, AMP-SCZ).^67^

In conclusion, these data provide initial evidence as a proof-of-concept, suggesting that the PPS may be a promising tool for improving the detection of CHR-P individuals but the potential for predicting transition to psychosis may be more limited.

## Supplementary Material

Supplementary_materials_sbag088

## Collaborators

Maria Calem,^1^ Stefania Tognin,^1^ Gemma Modinos,^2^ Eva Velthorst,^3, 4^ Tamar C. Kraan,^3^ Daniella S. van Dam, ^3^ Nadine Burger,^5^ G Paul Amminger,^6^ Christos Pantelis,^7^ Athena Politis,^6^ Joanne Goodall,^6^ Stefan Borgwardt,^8^ Ary Gadelha,^9^ Elisa Brietzke,^10^ Graccielle Asevedo,^9^ Elson Asevedo,^9^ Andre Zugman,^9^ Tecelli Domínguez-Martínez,^11^ Anna Racioppi,^12^ Thomas R Kwapil,^13^ Manel Monsonet,^12^ Lidia Hinojosa,^12^ Mathilde Kazes,^14^ Claire Daban,^14^ Julie Bourgin,^14^ Olivier Gay,^14^ Célia Mam-Lam-Fook,^14^ Dorte Nordholm,^15^ Lasse Randers,^15^ Kristine Krakauer,^15^ Louise Glenthøj,^15^ Birte Glenthøj,^16^ Dominika Gebhard,^17^ Julia Arnhold,^18^ Joachim Klosterkötter,^17^ Iris Lasser,^19^ Bernadette Winklbaur,^19^ Philippe A Delespaul^20, 21^

1. Department of Psychosis Studies, Institute of Psychiatry, Psychology & Neuroscience, King’s College London, De Crespigny Park, Denmark 458 Hill, London, United Kingdom SE5 8AF.
2. Department of Psychological Medicine, Institute of Psychiatry, Psychology & Neuroscience, King’s College London, De Crespigny Park, Denmark Hill, 456 London, United Kingdom SE5 8AF.
3. AMC, Academic Psychiatric Centre, Department Early Psychosis, Meibergdreef 5, 1105 AZ Amsterdam, The Netherlands.
4. Icahn School of Medicine at Mount Sinai, department of Psychiatry, 1425 Madison Ave, New York, NY 10029.
5. Parnassia Psychiatric Institute, Department of Psychosis Research, Zoutkeetsingel 40, 2512 HN The Hague, The Netherlands.
6. Centre for Youth Mental Health, University of Melbourne, 35 Poplar Road (Locked Bag 10), Parkville, Victoria 4 853 052, Australia.
7. Melbourne Neuropsychiatry Centre, University of Melbourne & Melbourne Health, Carlton South, Vic, Australia
8. Translational Psychiatry, Department of Psychiatry and Psychotherapy, University of Luebeck, Ratzeburger Allee 160, Lübeck, 23 562, Germany
9. Depto Psiquiatria, Escola Paulista de Medicina, Universidade Federal de São Paulo – UNIFESP.
10. Departament de Psicologia Clínica i de la Salut (Universitat Autònoma de Barcelona), Fundació Sanitària Sant Pere Claver (Spain), Spanish Mental Health Research Network (CIBERSAM).
11. CONACYT-Dirección de Investigaciones Epidemiológicas y Psicosociales, Instituto Nacional de Psiquiatría Ramón de la Fuente Muñiz (México).
12. Departament de Psicologia Clínica i de la Salut (Universitat Autònoma de Barcelona).
13. Department of Psychology, University of Illinois at Urbana-Champaign (USA).
14. University Paris Cité, GHU Paris Psychiatrie et Neurosciences, C’JAAD, Pôle PEPIT, Inserm UMR1266, Institut de Psychiatrie (CNRS 3557) Paris, France 18.
15. Mental Health Center Copenhagen and Center for Clinical Intervention and Neuropsychiatric Schizophrenia Research, CINS, Mental Health Center Glostrup, Mental Health Services in the Capital Region of Copenhagen, University of Copenhagen.
16. Centre for Neuropsychiatric Schizophrenia Research (CNSR) & Centre for Clinical Intervention and Neuropsychiatric Schizophrenia Research (CINS), Mental Health Centre Glostrup, University of Copenhagen, Glostrup, Denmark
17. Department of Psychiatry and Psychotherapy, University of Cologne, Cologne, Germany.
18. Psyberlin, Berlin, Germany.
19. Medical University of Vienna, Department of Psychiatry and Psychotherapy.
20. Department of Psychiatry and Neuropsychology, School for Mental Health and Neuroscience, Maastricht University Medical Centre, P.O. Box 616, 6200 MD 464 Maastricht, The Netherlands.
21. Mondriaan Mental health Trust, P.O. Box 4436 CX Heerlen, The Netherlands.
