# Supplementary material for: Psychosis Polyrisk Score and Polygenic Risk Score to Improve Detection and Prognosis in Individuals at Clinical High Risk for Psychosis: Model Development and Internal Validation
Source: Schizophr Bull. 2026 Aug 24;52(5):sbag088. doi: 10.1093/schbul/sbag088 (PMC13502905; doi:10.1093/schbul/sbag088)
Supplement: Supplementary file 1 [file supplementary_materials_sbag088.docx]

**SUPPLEMENTARY MATERIAL**

Oliver D, Arribas M, Logeswaran Y et al. Psychosis Polyrisk Score (PPS) and polygenic risk score to improve detection and prognosis in individuals at clinical high risk for psychosis: model development and internal validation

**eMethods 1** Recruitment, inclusion/exclusion criteria and ethics statement for the EU-GEI study

**eMethods 2** List of all included risk/protective factors in PPS

**eTable 1** List of included factors, along with their definitions and cut-offs

**eMethods 3** Sources of external data sources

**eTable 2** TRIPOD+AI Checklist

**eFigure 1** Data missingness across PPS factors

**eMethods 4** Genotyping methods

**eMethods 5** Hyperparameter tuning and imputation

**eMethods 6** Decision curve analysis

**eFigure 2** Cumulative incidence of psychosis in CHR-P individuals

**eTable 3** Results of univariate associations for detection

**eTable 4** Results of univariate associations for prognosis

**eTable 5** Coefficients from detection logistic regression models including PPS predictors, PRS and combined predictors (PPS+PRS)

**eTable 6** Variable importance from detection random forest models including PPS predictors, PRS and combined predictors (PPS+PRS)

**eFigure 3** Calibration plots for logistic regression models (detection)

**eFigure 4** Calibration plots for random forest models (detection)

**eTable 7** Decision curve analyses for detection models

**eTable 8** Coefficients from prognosis Cox proportional hazards models combining clinical predictors, PPS predictors and PRS

**eTable 9** Variable importance from prognosis random survival forest models combining clinical predictors, PPS predictors and PRS

**eFigure 5** Calibration plots for Cox proportional hazards models (prognosis)

**eFigure 6** Calibration plots for random survival forest models (prognosis)

**eTable 10** Sensitivity analyses results

**eTable 11** Remission models

**eResults 1** Post hoc sample size calculation

**eMethods 1** Recruitment, inclusion/exclusion criteria and ethics statement for the EU-GEI study

CHR-P individuals were enrolled from eleven sites in Europe (Amsterdam, Barcelona, Basel, Cologne, Copenhagen, Den Haag, London, Paris, Vienna), Australia (Melbourne) and South America (São Paulo). HCs were recruited from four of the sites: Amsterdam, Den Haag, London and Melbourne.

CHR-P criteria were ascertained using the Comprehensive Assessment of At-Risk Mental States (CAARMS)^1^, a psychometric instrument that assesses attenuated psychotic symptomatology. Age was not restricted as some local clinical services used different entry requirements. 95.0% participants were 16-35 years old with a minority of participants who were slightly older (0.9%) or younger (4.1%). Exclusion criteria were: i) previous diagnosis of a psychotic disorder, as defined by the Structural Clinical Interview for DSM-IV^2^; ii) exceeding the psychosis threshold or antipsychotic treatment threshold defined by the CAARMS^1^; iii) an estimated IQ<60 as measured by the shortened WAIS^3^; iv) being unwilling to give a blood or saliva sample for genetic analysis. In addition, CHR-P subjects were excluded if their psychotic symptoms could be explained by an organic disorder or substance use, and HCs were excluded if they met CAARMS criteria for the CHR-P state.

Depression and anxiety disorder diagnoses were assessed with the Structured Clinical Interview for DSM-IV. All procedures were approved by research ethics committees at each site and complied with the ethical standards of the relevant national and institutional committees on human experimentation and with the Helsinki Declaration of 1975, as revised in 2008. Written, informed consent was provided by all participants.

**eMethods 2** List of all included risk/protective factors in PPS. Those assessed in this study are presented in **bold**.

We considered 17 factors that met the highest hierarchy of evidence (i.e. class I–III) for association with psychotic disorders, as detailed in an umbrella review of risk and protective factors for psychosis^4^. We then excluded those factors (n=6) that could not easily be measured at scale due to cost (Toxoplasma Gondii IgG), extended assessment time (premorbid IQ) or limited reliability of self-report (olfactory identification ability, minor physical anomalies). We also excluded the CHR-P state to investigate the PPS independently from this construct. This left 12 remaining class I-III risk factors. In addition to these risk factors, we additionally included some class IV risk factors that either overlapped with included factors or could be recorded at low cost, high reliability and limited assessment time (n=11).

We then excluded those factors (n=6) that could not easily be measured at scale due to cost (Toxoplasma Gondii IgG), extended assessment time (premorbid IQ) or limited reliability of self-report (olfactory identification ability, minor physical anomalies). We also excluded the CHR-P state to investigate the PPS independently from this construct. This left 12 remaining class I-III risk factors.

Simple adaptations were added to the model to account for interdependencies in exposures, such as the multiple risk/protective factors associated with immigration status. For example, individuals cannot be exposed to certain immigration-based risk/protective factors in conjunction with each other. Immigrants cannot be both first generation and second-generation, and North African immigrants have to be either first- or second-generation immigrants. We combined these factors following this logic and assuming that the proportion and extra risk of North African immigrants is similar in first- and second-generation immigrants^5^. Factors related to ethnicity have similar logical dependencies between them, e.g. Black Caribbean is a non-White ethnicity, and individuals cannot be from a low ethnic density area, from a medium density area and from a high ethnic density area at the same time. We combined these factors again following this logic and assumed that the proportion and extra risk of Black Caribbean individuals between non-White ethnicity individuals is similar in low, medium and high ethnic density areas. Limitations of the PPS were previously presented^6,7^.

Of the 21 previously defined PPS factors, data were not available for hearing problems in the past 12 months. Moreover, all participants endorsed paternal ages <35 and Black participants all lived in low ethnic density areas, meaning variance was zero, so these factors were dropped.

Data on ethnic density, pollution and urbanicity were generated through area-level data linked to participants’ home addresses.

We did not use the weightings associated with the original PPS factors, as the associated psychosis risk in CHR-P individuals likely differs to the generated equivalent Odds Ratios from the umbrella review^4^.

*Class I*

1. **Black‐Caribbean ethnicity in England (and Black African ethnicity in England – class IV)**

*Class II*

2. **Ethnic minority in low ethnic density area**
3. **Second generation immigrants**
4. **Trait anhedonia**

*Class III*

5. **Childhood trauma**
6. **Ethnic minority in high ethnic density area**
7. **First generation immigrants**
8. **Non‐right handedness**
9. **North African immigrants in Europe**
10. **Urbanicity**

*Class IV*

11. **Adult life events**
12. **Other ethnicity (Asian/Mixed/Other White ethnicity in England)**
13. **Heavy cannabis use**
14. **Low paternal socio‐economic status**
15. **Parental severe mental illness**
16. **Tobacco use**
17. **Pollution**
18. **Male & aged 25-35 years old**

| **eTable 1** List of included factors, along with their definitions and cut-offs | | | |
| --- | --- | --- | --- |
| **Risk factor assessed (Classification of evidence; Meta-analytical reference)** | **Assessment tool; Reference** | **Scoring (Classification of evidence; meta-analytical reference)** | **Cutoff** |
| Age/Gender^3,4^ (IV) |  |  | Aged 25-35 & Male |
| Non-right-handedness^5^ (III) |  |  |  |
| Pollution^6^ (IV) |  | Local census data | 90^th^ percentile of per capita emissions at local authority level |
| Urbanicity^7^ (III) |  | Local census data | 300 people per square kilometre (km^2^) or 3 people per hectare |
| Ethnic density |  | Local census data | Low: Lowest 33^rd^ percentile  Medium: Middle 33^rd^ percentile  High: Highest 33^rd^ percentile |
| Black Ethnicity^8^ (I) |  | In low ethnic density area^9^ (II) |  |
|  |  | In medium ethnic density area |  |
|  |  | In high ethnic density area^9^ (III) |  |
| Other Ethnicity^4^ (IV) |  | In low ethnic density area^9^ (II) |  |
|  |  | In medium ethnic density area |  |
|  |  | In high ethnic density area^9^ (III) |  |
| White |  |  |  |
| Not immigrant |  |  |  |
| 1st generation immigrant^10^ (III) |  | From North Africa^11^ (III) |  |
|  |  | From other regions |  |
| 2nd generation immigrant^10^ (II) |  | From North Africa^11^ (III) |  |
|  |  | From other regions |  |
| Paternal age <35 |  |  |  |
| Paternal age > 35^12^ (IV) |  |  |  |
| Paternal age > 45^12^ (IV) |  |  |  |
| Paternal SES^13^ (IV) |  |  |  |
| Parental Severe Mental Illness^15^ (IV) | Family Interview for Genetic Studies [FIGS]^16^ |  | ≥1 |
| Adult Life Events^17^ (IV) | Life Threatening Events Questionnaire [LTE-Q]^18^ |  | >0 |
| Tobacco use^19^ (IV) |  |  | Daily smoker |
| Heavy cannabis use^20^ (IV) | Cannabis Experience Questionnaire |  | More than once a week or every day |
| Childhood Trauma^21^ (III) | Childhood Trauma Questionnaire Short Form [CTQ-SF]^22^ | *Never true = 1, rarely true = 2, sometimes true = 3, often true = 4, Very often true =5. None = 5-40, Low = 41-55; Moderate = 56-72; Severe = 73+* | Moderate and above |
| Trait Anhedonia^23^ (II) | Comprehensive Assessment of At Risk Mental States [CAARMS] |  | Severity ≥3 |

**eMethods 3** Sources of linked data

**Australia**

- [Pollution](https://data.gov.au/dataset/ds-dga-77726fe7-ac78-4e4d-a8f7-05c55b417858/details?q=emissions) (state level)
- [Urbanicity](https://www.abs.gov.au/AUSSTATS/abs@.nsf/DetailsPage/3218.02016-17?OpenDocument)
- Ethnic density **-** Australian Bureau of Statistics (state level)

**Austria**

- [Pollution](https://aqicn.org/)
- [Population density](https://www.citypopulation.de/en/austria/cities/)
- [Ethnic density](https://en.wikipedia.org/wiki/Demographics_of_Austria)

**Belgium**

- [Pollution](https://aqicn.org/)
- [Population density](https://statbel.fgov.be/en/themes/population)
- Ethnic density – not available

**Brazil**

- [Pollution](https://aqicn.org/)
- [Population density](https://www.ibge.gov.br/en/cities-and-states/sp.html)
- [Ethnicity density](https://cidades.ibge.gov.br/brasil/sp/pesquisa/23/25888?detalhes=true)

**Denmark**

- [Pollution](https://aqicn.org/)
- [Population density](https://www.statbank.dk/10021)
- [Ethnic density](https://www.statbank.dk/10021)

**France**

- [Pollution](https://aqicn.org/)
- [Population density](https://www.ined.fr/en/everything_about_population/data/france/population-structure/regions_departments/#r151)
- Ethnic density – not available

**Germany**

- [Pollution](https://aqicn.org/)
- [Population density](https://www-genesis.destatis.de/gis/genView?GenMLURL=https://www-genesis.destatis.de/regatlas/AI002-1.xml&CONTEXT=REGATLAS01)
- Ethnic density – not available

**Netherlands**

- [Pollution](https://aqicn.org/)
- [Population density](https://opendata.cbs.nl/statline/#/CBS/nl/dataset/03759NED/table?ts=1551192262284)
- [Ethnic density](https://www.amsterdam.info/netherlands/population/#:~:text=Ethnic%20groups%3A%20Over%2081%2C7,Indonesians)%20(1999%20est.))

**Spain**

- [Pollution](https://aqicn.org/)
- [Population density](https://www.barcelona.de/en/barcelona-figueres.html)
- [Ethnic density](https://rm.coe.int/CoERMPublicCommonSearchServices/DisplayDCTMContent?documentId=09000016802ff6f2)

**Switzerland**

- [Pollution](https://aqicn.org/)
- [Population density](https://www.bfs.admin.ch/bfs/en/home/statistics/regional-statistics/regional-portraits-key-figures/cantons/basel-stadt.html)
- [Ethnic density](https://en.wikipedia.org/wiki/Demographics_of_Switzerland#Demographic_statistics)

**UK**

- [Pollution](https://www.ons.gov.uk/census)
- [Population density](https://www.ons.gov.uk/census)
- [Ethnic density](https://www.ons.gov.uk/census)

| **eTable 2** TRIPOD+AI Checklist | | | | |
| --- | --- | --- | --- | --- |
| **Section/Topic Item Development Checklist item**  **/ evaluation**^1^ | | | | **Reported on page** |
| **TITLE** | | | |  |
| *Title* | 1 | D;E | Identify the study as developing or evaluating the performance of a multivariable prediction model, the target population, and the outcome to be predicted | 1 |
| **ABSTRACT** | | | | |
| *Abstract* | 2 | D;E | See TRIPOD+AI for Abstracts checklist | 3 |
| **INTRODUCTION** | | | | |
| *Background* | 3a | D;E | Explain the healthcare context (including whether diagnostic or prognostic) and rationale for developing or evaluating the prediction model, including references to existing models | 6 |
|  | 3b | D;E | Describe the target population and the intended purpose of the prediction model in the context of the care pathway, including its intended users (e.g., healthcare professionals, patients, public) | 6 |
|  | 3c | D;E | Describe any known health inequalities between sociodemographic groups | N/A |
| *Objectives* | 4 | D;E | Specify the study objectives, including whether the study describes the development or validation of a prediction model (or both) | 6 |
| **METHODS** | | | | |
| *Data* | 5a | D;E | Describe the sources of data separately for the development and evaluation datasets (e.g., randomised trial, cohort, routine care or registry data), the rationale for using these data, and representativeness of the data | 7 |
|  | 5b | D;E | Specify the dates of the collected participant data, including start and end of participant accrual; and, if applicable, end of follow-up | 7 |
| *Participants* | 6a | D;E | Specify key elements of the study setting (e.g., primary care, secondary care, general population)  including the number and location of centres | eMethods 1 |
|  | 6b | D;E | Describe the eligibility criteria for study participants | eMetthods 1 |
|  | 6c | D;E | Give details of any treatments received, and how they were handled during model development or evaluation, if relevant | N/A |
| *Data preparation* | 7 | D;E | Describe any data pre-processing and quality checking, including whether this was similar across  relevant sociodemographic groups | eMethods 3 |
| *Outcome* | 8a | D;E | Clearly define the outcome that is being predicted and the time horizon, including how and when assessed, the rationale for choosing this outcome, and whether the method of outcome assessment is  consistent across sociodemographic groups | 8 |
|  | 8b | D;E | If outcome assessment requires subjective interpretation, describe the qualifications and demographic characteristics of the outcome assessors | N/A |
|  | 8c | D;E | Report any actions to blind assessment of the outcome to be predicted | N/A |
| *Predictors* | 9a | D | Describe the choice of initial predictors (e.g., literature, previous models, all available predictors) and  any pre-selection of predictors before model building | 7-8; eMethods 2-3; eTable 1 |
|  | 9b | D;E | Clearly define all predictors, including how and when they were measured (and any actions to blind assessment of predictors for the outcome and other predictors) | 7-8; eMethods 2-3; eTable 1 |
|  | 9c | D;E | If predictor measurement requires subjective interpretation, describe the qualifications and demographic characteristics of the predictor assessors | eMethods 1-2 |
| *Sample size* | 10 | D;E | Explain how the study size was arrived at (separately for development and evaluation), and justify that  the study size was sufficient to answer the research question. Include details of any sample size calculation | eResults 1 |
| *Missing data* | 11 | D;E | Describe how missing data were handled. Provide reasons for omitting any data | eMethods 5 |
| *Analytical methods* | 12a | D | Describe how the data were used (e.g., for development and evaluation of model performance) in the analysis, including whether the data were partitioned, considering any sample size requirements | 8-9; eMethods 5 |
|  | 12b | D | Depending on the type of model, describe how predictors were handled in the analyses (functional form,  rescaling, transformation, or any standardisation). | N/A |
|  | 12c | D | Specify the type of model, rationale^2^, all model-building steps, including any hyperparameter tuning,  and method for internal validation | 8-9; eMethods 5 |
|  | 12d | D;E | Describe if and how any heterogeneity in estimates of model parameter values and model performance was handled and quantified across clusters (e.g., hospitals, countries). See TRIPOD-Cluster for  additional considerations^3^ | 10 |
|  | 12e | D;E | Specify all measures and plots used (and their rationale) to evaluate model performance (e.g., discrimination, calibration, clinical utility) and, if relevant, to compare multiple models | 9-10 |
|  | 12f | E | Describe any model updating (e.g., recalibration) arising from the model evaluation, either overall or for particular sociodemographic groups or settings | N/A |
|  | 12g | E | For model evaluation, describe how the model predictions were calculated (e.g., formula, code, object, application programming interface) | 8 |
| *Class imbalance* | 13 | D;E | If class imbalance methods were used, state why and how this was done, and any subsequent methods to recalibrate the model or the model predictions | 9 |
| *Fairness* | 14 | D;E | Describe any approaches that were used to address model fairness and their rationale | N/A |
| *Model output* | 15 | D | Specify the output of the prediction model (e.g., probabilities, classification). Provide details and  rationale for any classification and how the thresholds were identified | 9 |

^1^ D=items relevant only to the development of a prediction model; E=items relating solely to the evaluation of a prediction model; D;E=items applicable to both the development and evaluation of a prediction model

^2^ Separately for all model building approaches.

^3^ TRIPOD-Cluster is a checklist of reporting recommendations for studies developing or validating models that explicitly account for clustering or explore heterogeneity in model performance (eg, at different hospitals or centres). Debray et al, BMJ 2023; 380: e071018 [DOI: 10.1136/bmj-2022-071018]

| *Training versus*  *evaluation* | 16 | D;E | Identify any differences between the development and evaluation data in healthcare setting, eligibility  criteria, outcome, and predictors | N/A |
| --- | --- | --- | --- | --- |
| *Ethical approval* | 17 | D;E | Name the institutional research board or ethics committee that approved the study and describe the participant-informed consent or the ethics committee waiver of informed consent | eMethods 1 |
| **OPEN SCIENCE** | | | | |
| *Funding* | 18a | D;E | Give the source of funding and the role of the funders for the present study | 15 |
| *Conflicts of interest* | 18b | D;E | Declare any conflicts of interest and financial disclosures for all authors | 16 |
| *Protocol* | 18c | D;E | Indicate where the study protocol can be accessed or state that a protocol was not prepared | Not prepared |
| *Registration* | 18d | D;E | Provide registration information for the study, including register name and registration number, or state  that the study was not registered | N/A |
| *Data sharing* | 18e | D;E | Provide details of the availability of the study data | N/A |
| *Code sharing* | 18f | D;E | Provide details of the availability of the analytical code^4^ | 8 |
| **PATIENT & PUBLIC INVOLVEMENT** | | | | |
| *Patient & Public Involvement* | 19 | D;E | Provide details of any patient and public involvement during the design, conduct, reporting, interpretation, or dissemination of the study or state no involvement. | No involvement |
| **RESULTS** | | | | |
| *Participants* | 20a | D;E | Describe the flow of participants through the study, including the number of participants with and without the outcome and, if applicable, a summary of the follow-up time. A diagram may be helpful. | 10 |
|  | 20b | D;E | Report the characteristics overall and, where applicable, for each data source or setting, including the key dates, key predictors (including demographics), treatments received, sample size, number of outcome events, follow-up time, and amount of missing data. A table may be helpful. Report any  differences across key demographic groups. | Table 1 |
|  | 20c | E | For model evaluation, show a comparison with the development data of the distribution of important predictors (demographics, predictors, and outcome). | N/A |
| *Model development* | 21 | D;E | Specify the number of participants and outcome events in each analysis (e.g., for model development, hyperparameter tuning, model evaluation) | 10 |
| *Model specification* | 22 | D | Provide details of the full prediction model (e.g., formula, code, object, application programming interface) to allow predictions in new individuals and to enable third-party evaluation and implementation, including any restrictions to access or re-use (e.g., freely available, proprietary)^5^ | eTables 5-6, 8-9 |
| *Model performance* | 23a | D;E | Report model performance estimates with confidence intervals, including for any key subgroups (e.g., sociodemographic). Consider plots to aid presentation. | 11-12; Figure 2; Table 2 |
|  | 23b | D;E | If examined, report results of any heterogeneity in model performance across clusters. See TRIPOD  Cluster for additional details^3^. | N/A |
| *Model updating* | 24 | E | Report the results from any model updating, including the updated model and subsequent performance | N/A |
| **DISCUSSION** | | | | |
| *Interpretation* | 25 | D;E | Give an overall interpretation of the main results, including issues of fairness in the context of the  objectives and previous studies | 12-14 |
| *Limitations* | 26 | D;E | Discuss any limitations of the study (such as a non-representative sample, sample size, overfitting, missing data) and their effects on any biases, statistical uncertainty, and generalizability | 14 |
| *Usability of the model in the context of current care* | 27a | D | Describe how poor quality or unavailable input data (e.g., predictor values) should be assessed and handled when implementing the prediction model | 14 |
|  | 27b | D | Specify whether users will be required to interact in the handling of the input data or use of the model,  and what level of expertise is required of users | 14 |
|  | 27c | D;E | Discuss any next steps for future research, with a specific view to applicability and generalizability of  the model | 12-13 |

From: Collins GS, Moons KGM, Dhiman P, et al. *BMJ* 2024;385:e078378. doi:10.1136/bmj-2023-078378

^4^ This relates to the analysis code, for example, any data cleaning, feature engineering, model building, evaluation.

^5^ This relates to the code to implement the model to get estimates of risk for a new individual.

**eFigure 1** Data missingness across PPS factors

**eMethods 4** Genotyping methods

These samples were genotyped at the MRC Centre for Neuropsychiatric Genetics and Genomics in Cardiff (UK) by a custom Illumina HumanCoreExome-24 BeadChip genotyping array, including 570,038 genetic variants. Genetic variants were imputed using the HRC as reference (version r1.1 2016) through the Michigan Imputation Server. Variant QC was conducted in Plink v2.0 and excluded variants with missing genotype rates > 0.1, Hardy-Weinberg Equilibrium p<1e-50 and samples with call rate <60%. After restricting to common genetic variants with minor allele frequency (MAF) of > 0.01, the genetic sample of EU-GEI consisted of 385 individuals and 9,308,829 single nucleotide polymorphisms (SNPs).

A Principal Component Analysis was performed on the full set of variants and samples using Plink (version 2.0) and the first 10 principal components (PCs) were retained to control for the effects of population stratification.

PRS-SCZ was built using the software SBayesRC ^8^on the latest genome-wide association study (GWAS) results for schizophrenia^9^, using the European ancestry samples only. The PRS weights for the set of 6,677,769 genetic variants overlapping with the EU-GEI imputed sample was used to calculate a PRS for schizophrenia using Plink (version 2.0).

**eMethods 5** Hyperparameter tuning and imputation

Hyperparameters were tuned using random grid search within the inner folds of the cross-validation structure. These hyperparameters included lambda for LASSO and mtry (ranging from 3 to total number of predictors), minimum node size (ranging from 1 to 10% of the length of the training data) and number of trees (25, 50, 100, 250 or 500) for random forest models. The hyperparameters from the best performing model within the inner folds, as defined by the highest F1 score in detection models and highest Harrell’s C in prognosis models, were then used to refit on all training data and tested on the outer fold data.

Missing data were imputed following investigation of missingness patterns using the naniar package (version 1.1.0; eFigure 1). Random forest imputation (missForest version 1.5; default parameters) was preferred with multiple imputation using chained equations (mice version 3.16.0; default parameters) if model did not converge.

**eMethods 6** Decision curve analysis

Net benefit is a metric of true positives minus false positives, and is calculated as:

sensitivity × prevalence – (1 – specificity) × (1 – prevalence) × w

where w is the outcome odds at a given risk threshold^10^. The risk threshold is the amount of tolerable risk before an intervention is deemed necessary. Net benefit incorporates the consequences of the decisions made on the basis of an algorithm, and is therefore preferable to related measures such as sensitivity and specificity alone^10^. A decision curve plot was used to visualise the net benefit of each detection and prognosis model over varying risk thresholds compared with screening all individuals or none. Standardised net benefit was calculated as the net benefit divided by the prevalence, showing the percentage increase in net benefit at a given risk threshold. Classical decision theory proposes that at a chosen risk threshold, the choice with the greatest net benefit should be preferred^11^.

**eFigure 2** Cumulative incidence (Kaplan–Meier failure function) for risk of development of psychosis among CHR-P individuals (n=344). Time on the figure is presented in years. The cumulative incidence of psychosis was 7.6 per 10,000 people (95%CI: 4.7-10.4, 314 individuals still at risk) at 6 months, 13.0 (95%CI: 9.4-16.6, 281 individuals still at risk) at 12 months, 15.9 (95%CI: 11.8-19.8, 229 individuals still at risk) at 18 months and 17.8 (95%CI: 13.4-21.9, 208 individuals still at risk) at 24 months and 23.2 (95%CI: 14.7-30.7, 24 individuals still at risk) at 36 months. The last transition to psychosis was at 1,022 days when 24 individuals were still at risk.

| **eTable 3** Results of univariate analyses for detection models. Significant findings are presented in **bold**. | | | | | |
| --- | --- | --- | --- | --- | --- |
| **Predictor** | **Coefficient** | **SE** | **OR** | **Z** | **p-value** |
| Age/Gender | 0.092 | 0.390 | 1.096 | 0.235 | 0.814 |
| **Adult life events** | **1.041** | **0.311** | **2.833** | **3.352** | **0.001** |
| **Childhood trauma** | **2.671** | **0.809** | **14.450** | **3.300** | **0.001** |
| Ethnicity: Black in low ethnic density area | 16.501 | 1769.258 | 14670624.940 | 0.009 | 0.993 |
| Ethnicity: Other in high ethnic density area | -0.613 | 0.501 | 0.542 | -1.222 | 0.222 |
| Ethnicity: Other in low ethnic density area | 2.113 | 1.085 | 8.276 | 1.949 | 0.051 |
| Ethnicity: Other in medium ethnic density area | 16.501 | 959.515 | 14670624.940 | 0.017 | 0.986 |
| Ethnicity: White | 0.707 | 0.403 | 2.028 | 1.755 | 0.079 |
| **Heavy cannabis use** | **1.181** | **0.382** | **3.259** | **3.093** | **0.002** |
| Immigration: 1^st^ generation immigrant from North Africa | 12.933 | 882.743 | 413755.273 | 0.015 | 0.988 |
| Immigration: 1^st^ generation immigrant from other regions | -0.203 | 0.299 | 0.816 | -0.680 | 0.497 |
| Immigration: 2^nd^ generation immigrant from North Africa | 13.945 | 650.874 | 1137974.813 | 0.021 | 0.983 |
| Immigration: 2^nd^ generation immigrant from other regions | 0.041 | 0.306 | 1.042 | 0.135 | 0.893 |
| Non-right-handedness | 0.747 | 0.674 | 2.110 | 1.108 | 0.268 |
| **Parental severe mental illness** | **1.666** | **0.323** | **5.289** | **5.164** | **<0.001** |
| Paternal socioeconomic status | 0.620 | 0.408 | 1.859 | 1.518 | 0.129 |
| Pollution | -16.034 | 678.479 | 0.000 | -0.024 | 0.981 |
| **Tobacco use** | **1.121** | **0.301** | **3.067** | **3.721** | **<0.001** |
| **Trait anhedonia** | **2.518** | **0.393** | **12.408** | **6.407** | **<0.001** |
| Urbanicity | -1.052 | 1.053 | 0.349 | -1.000 | 0.317 |
| **PRS** | **0.866** | **0.342** | **2.377** | **2.532** | **0.011** |
| **Abbreviations**: OR, Odds Ratio; PRS, polygenic risk score for schizophrenia | | | | | |

| **eTable 4** Results of univariate analyses for prognosis models. Significant findings are presented in **bold**. | | | | | |
| --- | --- | --- | --- | --- | --- |
| **Predictor** | **Coefficient** | **SE** | **OR** | **Z** | **p-value** |
| Age/Gender | 0.175 | 0.347 | 1.192 | 0.506 | 0.613 |
| Adult life events | 0.347 | 0.419 | 1.415 | 0.829 | 0.407 |
| Childhood trauma | 0.072 | 0.299 | 1.074 | 0.240 | 0.810 |
| Ethnicity: Black in low ethnic density area | -16.357 | 3317.152 | 0.000 | -0.005 | 0.996 |
| Ethnicity: Other in high ethnic density area | -0.940 | 0.803 | 0.391 | -1.171 | 0.242 |
| Ethnicity: Other in low ethnic density area | -0.668 | 0.692 | 0.513 | -0.966 | 0.334 |
| Ethnicity: Other in medium ethnic density area | 0.322 | 0.587 | 1.380 | 0.548 | 0.584 |
| Ethnicity: White | -0.310 | 0.409 | 0.733 | -0.758 | 0.448 |
| Handedness | -0.790 | 0.682 | 0.454 | -1.159 | 0.247 |
| Heavy cannabis use | 0.162 | 0.271 | 1.175 | 0.597 | 0.550 |
| Immigration: 1^st^ generation immigrant from North Africa | -14.006 | 2483.634 | 0.000 | -0.006 | 0.996 |
| Immigration: 1^st^ generation immigrant from other regions | 0.414 | 0.278 | 1.513 | 1.492 | 0.136 |
| Immigration: 2^nd^ generation immigrant from North Africa | 0.322 | 1.009 | 1.380 | 0.320 | 0.749 |
| Immigration: 2^nd^ generation immigrant from other regions | 0.276 | 0.278 | 1.317 | 0.993 | 0.321 |
| Parental severe mental illness | 0.002 | 0.303 | 1.002 | 0.005 | 0.996 |
| Paternal socioeconomic status | 0.008 | 0.329 | 1.008 | 0.026 | 0.980 |
| Pollution | 0.247 | 0.467 | 1.281 | 0.529 | 0.597 |
| Tobacco use | -0.179 | 0.259 | 0.836 | -0.690 | 0.490 |
| Trait anhedonia | 0.348 | 0.284 | 1.416 | 1.225 | 0.220 |
| Urbanicity | -0.224 | 0.594 | 0.799 | -0.377 | 0.706 |
| PRS | 0.472 | 0.319 | 1.603 | 1.481 | 0.139 |
| CAARMS | 0.004 | 0.026 | 1.004 | 0.159 | 0.874 |
| **GAF symptoms** | **-0.027** | **0.013** | **0.974** | **-2.052** | **0.040** |
| GAF disability | -0.013 | 0.011 | 0.987 | -1.174 | 0.241 |
| **Abbreviations**: CAARMS, Comprehensive Assessment of At Risk Mental State; GAF, Global Assessment of Functioning; HR, Hazard Ratio; PRS, polygenic risk score for schizophrenia | | | | | |

| **eTable 5** Coefficients from detection logistic regression models including PPS predictors, PRS and combined predictors (PPS+PRS) | | | |
| --- | --- | --- | --- |
| **Predictor** | **PPS** | **PRS** | **PPS+PRS** |
| (Intercept) | 1.187 | 1.705 | 0.890 |
| **PPS** |  |  |  |
| Adult life events | 0.228 | NA | 0.409 |
| Age/Gender | 0.351 | NA | 0.069 |
| Childhood trauma | 3.153 | NA | 2.943 |
| Ethnicity: Black in low ethnic density area | 2.174 | NA | 2.090 |
| Ethnicity: White | 0.376 | NA | 0.230 |
| Ethnicity: Other in low ethnic density area | 1.459 | NA | 1.626 |
| Ethnicity: Other in medium ethnic density area | 3.067 | NA | 2.960 |
| Ethnicity: Other in high ethnic density area | -2.431 | NA | -1.944 |
| Heavy cannabis use | 1.187 | NA | 1.253 |
| Immigration: 1^st^ generation immigrant from North Africa | NA | NA | NA |
| Immigration: 1^st^ generation immigrant from other regions | 0.593 | NA | 0.378 |
| Immigration: 2^nd^ generation immigrant from North Africa | 2.340 | NA | 1.524 |
| Immigration: 2^nd^ generation immigrant from other regions | 0.976 | NA | 0.984 |
| Non-right-handedness | NA | NA | NA |
| Parental severe mental illness | 1.191 | NA | 1.110 |
| Paternal socioeconomic status | 1.262 | NA | 0.959 |
| Pollution | -4.463 | NA | -3.989 |
| Tobacco | 0.746 | NA | 0.779 |
| Trait anhedonia | 3.039 | NA | 3.050 |
| Urbanicity | NA | NA | -0.011 |
| **PRS** |  |  |  |
| PRS | NA | 0.822 | 0.811 |
| **Abbreviations**: PRS, polygenic risk score for schizophrenia | | | |
|  |  |  |  |

| **eTable 6** Variable importance from detection random forest models including PPS predictors, PRS and combined predictors (PPS+PRS) | | | | | | | | | | |
| --- | --- | --- | --- | --- | --- | --- | --- | --- | --- | --- |
| **Ranking** | **PPS** | | | **PRS** | | **PPS+PRS** | | | |  |
|  | **Predictor** | **Importance (%)** | **Predictor** | | **Importance (%)** | | **Predictor** | **Importance (%)** |  |  |
| **1** | Anhedonia | 100.0 | PRS | | 100.0 | | Anhedonia | 100.0 |  |  |
| **2** | Parental severe mental illness | 71.3 | NA | | NA | | PRS | 82.0 |  |  |
| **3** | Non-right-handedness | 57.0 | NA | | NA | | Parental severe mental illness | 66.3 |  |  |
| **4** | Ethnicity: Other in high ethnic density area | 25.8 | NA | | NA | | Non-right-handedness | 40.7 |  |  |
| **5** | Childhood Trauma | 20.4 | NA | | NA | | Ethnicity: Other in high ethnic density area | 20.8 |  |  |
| **6** | Heavy cannabis use | 18.2 | NA | | NA | | Heavy cannabis use | 16.9 |  |  |
| **7** | Stressful Life Events | 13.7 | NA | | NA | | Childhood Trauma | 14.2 |  |  |
| **8** | Tobacco use | 12.1 | NA | | NA | | Stressful Life Events | 11.4 |  |  |
| **9** | Pollution | 10.6 | NA | | NA | | Tobacco use | 9.3 |  |  |
| **10** | Immigration: 1^st^ generation immigrant from other regions | 8.4 | NA | | NA | | Pollution | 7.8 |  |  |
| **11** | Gender: Male 25-35 | 8.1 | NA | | NA | | Immigration: 1^st^ generation immigrant from other regions | 6.3 |  |  |
| **12** | Ethnicity: White | 7.1 | NA | | NA | | Gender: Male 25-35 | 6.1 |  |  |
| **13** | Paternal socioeconomic status | 5.9 | NA | | NA | | Ethnicity: White | 5.7 |  |  |
| **14** | Urbanicity | 4.1 | NA | | NA | | Immigration: 2^nd^ generation immigrant from other regions | 5.2 |  |  |
| **15** | Immigration: 2^nd^ generation immigrant from other regions | 3.6 | NA | | NA | | Paternal socioeconomic status | 4.4 |  |  |
| **16** | Ethnicity: Other in medium ethnic density area | 2.3 | NA | | NA | | Urbanicity | 3.0 |  |  |
| **17** | Ethnicity: Other in low ethnic density area | 1.7 | NA | | NA | | Ethnicity: Other in medium ethnic density area | 2.8 |  |  |
| **18** | Immigration: 2^nd^ generation immigrant from North Africa | 0.1 | NA | | NA | | Ethnicity: Other in low ethnic density area | 1.2 |  |  |
| **19** | Immigration: 1^st^ generation immigrant from North Africa | 0.1 | NA | | NA | | Immigration: 2^nd^ generation immigrant from North Africa | 0.2 |  |  |
| **20** | Ethnicity: Black in low ethnic density area | 0 | NA | | NA | | Immigration: 1^st^ generation immigrant from North Africa | 0.0 |  |  |

**eFigure 3** Calibration plots for logistic regression models (detection). Each calibration plot is derived from all prediction data from test sets across our repeated, nested cross-validation framework. This may lead to narrower CIs than any single calibration plot. **A** PPS; **B** PRS; **C** PPS+PRS

**eFigure 4** Calibration plots for random forest models (detection). Each calibration plot is derived from all prediction data from test sets across our repeated, nested cross-validation framework. This may lead to narrower CIs than any single calibration plot. **A** PPS; **B** PRS; **C** PPS+PRS

**C**

**B**

**A**

**C**

**B**

**A**

| **eTable 7** Results of decision curve analyses for detection models | | | |
| --- | --- | --- | --- |
| **Condition** | **Threshold** | **Net Benefit** | **Standardised Net Benefit** |
| **Clinical Populations** | | | |
| PPS | 0 | 0.000 | 0.000 |
| PPS | 0.1 | **0.012** | **0.063** |
| PPS | 0.2 | **0.065** | **0.337** |
| PPS | 0.3 | **0.042** | **0.221** |
| PPS | 0.4 | **0.017** | **0.089** |
| PPS | 0.5 | -0.017 | -0.089 |
| PRS | 0 | 0.000 | 0.000 |
| PRS | 0.1 | 0.000 | 0.000 |
| PRS | 0.2 | -0.010 | -0.052 |
| PRS | 0.3 | -0.152 | -0.792 |
| PRS | 0.4 | -0.303 | -1.579 |
| PRS | 0.5 | -0.268 | -1.398 |
| PPS+PRS | 0 | 0.000 | 0.000 |
| PPS+PRS | 0.1 | **0.013** | **0.067** |
| PPS+PRS | 0.2 | **0.066** | **0.342** |
| PPS+PRS | 0.3 | **0.039** | **0.205** |
| PPS+PRS | 0.4 | **0.020** | **0.106** |
| PPS+PRS | 0.5 | -0.013 | -0.070 |
| **General Population** | | | |
| PPS | 0 | 0.0000 | 0.0000 |
| PPS | 0.01 | **0.0001** | **0.0047** |
| PPS | 0.02 | -0.0025 | -0.1471 |
| PPS | 0.03 | -0.0121 | -0.7138 |
| PPS | 0.04 | -0.0218 | -1.2796 |
| PPS | 0.05 | -0.0310 | -1.8262 |
| PPS | 0.06 | -0.0405 | -2.3797 |
| PPS | 0.07 | -0.0489 | -2.8745 |
| PPS | 0.08 | -0.0573 | -3.3727 |
| PPS | 0.09 | -0.0651 | -3.8280 |
| PPS | 0.1 | -0.0739 | -4.3482 |
| PRS | 0 | 0.0000 | 0.0000 |
| PRS | 0.01 | 0.0000 | 0.0000 |
| PRS | 0.02 | -0.0031 | -0.1801 |
| PRS | 0.03 | -0.0134 | -0.7884 |
| PRS | 0.04 | -0.0240 | -1.4093 |
| PRS | 0.05 | -0.0347 | -2.0433 |
| PRS | 0.06 | -0.0457 | -2.6909 |
| PRS | 0.07 | -0.0570 | -3.3523 |
| PRS | 0.08 | -0.0685 | -4.0281 |
| PRS | 0.09 | -0.0802 | -4.7188 |
| PRS | 0.1 | -0.092 | -5.425 |
| PPS+PRS | 0 | 0.0000 | 0.0000 |
| PPS+PRS | 0.01 | **0.0001** | **0.0068** |
| PPS+PRS | 0.02 | -0.0025 | -0.1488 |
| PPS+PRS | 0.03 | -0.0122 | -0.7176 |
| PPS+PRS | 0.04 | -0.0213 | -1.2511 |
| PPS+PRS | 0.05 | -0.0302 | -1.7757 |
| PPS+PRS | 0.06 | -0.0389 | -2.2895 |
| PPS+PRS | 0.07 | -0.0481 | -2.8287 |
| PPS+PRS | 0.08 | -0.0563 | -3.3138 |
| PPS+PRS | 0.09 | -0.0644 | -3.7865 |
| PPS+PRS | 0.1 | -0.072 | -4.215 |

| **eTable 8** Beta coefficients from prognosis Cox proportional hazards models including clinical predictors, PPS predictors, PRS and combined predictors. | | | | | | | | |
| --- | --- | --- | --- | --- | --- | --- | --- | --- |
| **Predictor** | **Clinical** | **PPS** | **PRS** | **Clinical+PPS** | **Clinical+PRS** | **PPS+PRS** | **Clinical+PPS+PRS** |  |
| **Clinical** |  |  |  |  |  |  |  |  |
| CAARMS | NA | NA | NA | NA | NA | NA | NA |  |
| GAF symptoms | -0.005 | NA | NA | -0.003 | -0.0201 | NA | -0.008 |  |
| GAF disability/impairment | NA | NA | NA | NA | -0.0008 | NA | NA |  |
| **PPS** |  |  |  |  |  |  |  |  |
| Adult life events | NA | NA | NA | NA | NA | NA | NA |  |
| Age/Gender | NA | 0.0007 | NA | NA | NA | NA | NA |  |
| Childhood trauma | NA | NA | NA | NA | NA | NA | NA |  |
| Ethnicity: Black in low ethnic density area | NA | NA | NA | NA | NA | NA | NA |  |
| Ethnicity: Black in high ethnic density area | NA | NA | NA | NA | NA | NA | NA |  |
| Ethnicity: White | NA | NA | NA | NA | NA | NA | NA |  |
| Ethnicity: Other in low ethnic density area | NA | NA | NA | NA | NA | NA | NA |  |
| Ethnicity: Other in medium ethnic density area | NA | NA | NA | NA | NA | NA | NA |  |
| Ethnicity: Other in high ethnic density area | NA | NA | NA | NA | NA | NA | NA |  |
| Heavy cannabis use | NA | NA | NA | NA | NA | NA | NA |  |
| Immigration: 1^st^ generation immigrant from North Africa | NA | NA | NA | NA | NA | NA | NA |  |
| Immigration: 1^st^ generation immigrant from other regions | NA | -0.0352 | NA | NA | NA | NA | -0.062 |  |
| Immigration: 2^nd^ generation immigrant from North Africa | NA | NA | NA | NA | NA | NA | NA |  |
| Immigration: 2^nd^ generation immigrant from other regions | NA | NA | NA | NA | NA | NA | NA |  |
| Non-right-handedness | NA | NA | NA | NA | NA | NA | NA |  |
| Parental severe mental illness | NA | NA | NA | NA | NA | NA | NA |  |
| Paternal socioeconomic status | NA | NA | NA | NA | NA | NA | NA |  |
| Pollution | NA | NA | NA | NA | NA | NA | NA |  |
| Tobacco | NA | NA | NA | NA | NA | NA | NA |  |
| Trait anhedonia | NA | NA | NA | NA | NA | NA | NA |  |
| Urbanicity | NA | NA | NA | NA | NA | NA | NA |  |
| **PRS** |  |  |  |  |  |  |  |  |
| PRS | NA | NA | 0.156 | NA | 0.3042 | 0.0713 | 0.173 |  |
| **Abbreviations**: CAARMS, Comprehensive Assessment for At Risk Mental States; GAF, Global Assessment of Functioning; PC, principal component; PRS, polygenic risk score for schizophrenia; REF, reference category | | | | | | | | |
|  |  |  |  |  |  |  |  |  |
|  |  |  |  |  |  |  |  |  |

| **eTable 9** Variable importance from prognosis random survival forest models including clinical predictors, PPS predictors, PRS and combined predictors. | | | | | | | |
| --- | --- | --- | --- | --- | --- | --- | --- |
| **Ranking** | **Clinical** | | | **PPS** | |  |  |
|  | **Predictor** | **Importance** | **Predictor** | | **Importance** | |  |
| **1** | GAF symptoms | 0.11 | Immigration: 1^st^ generation immigrant from other regions | | 0.042 | |  |
| **2** | GAF disability | 0.02 | Gender: Male 25-35 | | 0.033 | |  |
| **3** | CAARMS | 0.002 | Immigration: 2^nd^ generation immigrant from North Africa | | 0.022 | |  |
| **4** | NA | NA | Urbanicity | | 0.016 | |  |
| **5** | NA | NA | Anhedonia | | 0.014 | |  |
| **6** | NA | NA | Ethnicity: White | | 0.012 | |  |
| **7** | NA | NA | Stressful life events | | 0.012 | |  |
| **8** | NA | NA | Ethnicity: Other in medium ethnic density area | | 0.010 | |  |
| **9** | NA | NA | Ethnicity: Black in high ethnic density area | | 0.008 | |  |
| **10** | NA | NA | Paternal socioeconomic status | | 0.007 | |  |
| **11** | NA | NA | Pollution | | 0.001 | |  |
| **12** | NA | NA | Immigration: 2^nd^ generation immigrant from North Africa | | 0.001 | |  |
| **13** | NA | NA | Immigration: 1^st^ generation immigrant from North Africa | | 0.000 | |  |
| **14** | NA | NA | Ethnicity: Other in low ethnic density area | | -0.001 | |  |
| **15** | NA | NA | Ethnicity: Black in low ethnic density area | | -0.001 | |  |
| **16** | NA | NA | Ethnicity: Other in high ethnic density area | | -0.004 | |  |
| **17** | NA | NA | Childhood trauma | | -0.005 | |  |
| **18** | NA | NA | Parental severe mental illness | | -0.006 | |  |
| **19** | NA | NA | Gender: Male 25-35 | | -0.008 | |  |
| **20** | NA | NA | Tobacco use | | -0.019 | |  |

| **Ranking** | **PRS** | | | **Clinical+PPS** | |  |
| --- | --- | --- | --- | --- | --- | --- |
|  | **Predictor** | **Predictor** | **Predictor** | | **Importance** | |
| **1** | PRS | 0.059 | GAF symptoms | | 0.065 | |
| **2** | NA | NA | Immigration: 1^st^ generation immigrant from other regions | | 0.027 | |
| **3** | NA | NA | Childhood Trauma | | 0.013 | |
| **4** | NA | NA | Anhedonia | | 0.010 | |
| **5** | NA | NA | Ethnicity: White | | 0.010 | |
| **6** | NA | NA | GAF disability | | 0.009 | |
| **7** | NA | NA | Ethnicity: Other in high ethnic density area | | 0.008 | |
| **8** | NA | NA | Ethnicity: Other in medium ethnic density area | | 0.008 | |
| **9** | NA | NA | Stressful life events | | 0.008 | |
| **10** | NA | NA | Ethnicity: Black in high ethnic density area | | 0.002 | |
| **11** | NA | NA | Paternal socioeconomic status | | 0.002 | |
| **12** | NA | NA | Non-right-handedness | | 0.001 | |
| **13** | NA | NA | Immigration: 1^st^ generation immigrant from North Africa | | 0.000 | |
| **14** | NA | NA | Ethnicity: Other in low ethnic density area | | 0.000 | |
| **15** | NA | NA | Ethnicity: Black in low ethnic density area | | -0.001 | |
| **16** | NA | NA | Pollution | | -0.001 | |
| **17** | NA | NA | CAARMS | | -0.002 | |
| **18** | NA | NA | Immigration: 2^nd^ generation immigrant from North Africa | | -0.003 | |
| **19** | NA | NA | Urbanicity | | -0.003 | |
| **20** | NA | NA | Parental severe mental illness | | -0.004 | |

| **Ranking** | **Clinical+PRS** | | | **PPS+PRS** | | |  |
| --- | --- | --- | --- | --- | --- | --- | --- |
|  | **Predictor** | **Importance** | **Predictor** | | **Importance** |  |  |
| **1** | GAF symptoms | 0.098 | PRS | | 0.038 |  |  |
| **2** | CAARMS | 0.016 | Non-right-handedness | | 0.035 |  |  |
| **3** | GAF disability | 0.005 | Immigration: 1^st^ generation immigrant from other regions | | 0.026 |  |  |
| **4** | PRS | 0.003 | Anhedonia | | 0.025 |  |  |
| **5** | NA | NA | Stressful life events | | 0.018 |  |  |
| **6** | NA | NA | Immigration: 1^st^ generation immigrant from other regions | | 0.016 |  |  |
| **7** | NA | NA | Ethnicity: Other in medium ethnic density area | | 0.016 |  |  |
| **8** | NA | NA | Parental severe mental illness | | 0.013 |  |  |
| **9** | NA | NA | Ethnicity: Black in high ethnic density area | | 0.009 |  |  |
| **10** | NA | NA | Ethnicity: White | | 0.009 |  |  |
| **11** | NA | NA | Pollution | | 0.006 |  |  |
| **12** | NA | NA | Ethnicity: Other in high ethnic density area | | 0.004 |  |  |
| **13** | NA | NA | Urbanicity | | 0.004 |  |  |
| **14** | NA | NA | Paternal socioeconomic status | | 0.003 |  |  |
| **15** | NA | NA | Ethnicity: Other in low ethnic density area | | 0.002 |  |  |
| **16** | NA | NA | Ethnicity: Black in low ethnic density area | | 0.001 |  |  |
| **17** | NA | NA | Immigration: 1^st^ generation immigrant from North Africa | | 0.000 |  |  |
| **18** | NA | NA | Tobacco use | | 0.000 |  |  |
| **19** | NA | NA | Immigration: 2^nd^ generation immigrant from North Africa | | -0.002 |  |  |
| **20** | NA | NA | Gender: Male 25-35 | | -0.005 |  |  |

| **Clinical+PPS+PRS** | |  |
| --- | --- | --- |
| **Predictor** | **Importance** | |
| GAF symptoms | 0.070 | |
| Immigration: 1^st^ generation immigrant from other regions | 0.027 | |
| GAF disability | 0.013 | |
| Stressful life events | 0.012 | |
| Anhedonia | 0.010 | |
| Ethnicity: White | 0.009 | |
| Ethnicity: Other in high ethnic density area | 0.004 | |
| Handedness | 0.004 | |
| Childhood trauma | 0.003 | |
| Immigration: 2^nd^ generation immigrant from North Africa | 0.003 | |
| PRS | 0.003 | |
| Ethnicity: Other in low ethnic density area | 0.002 | |
| Parental severe mental illness | 0.001 | |
| CAARMS | 0.001 | |
| Ethnicity: Black in low ethnic density area | 0.000 | |
| Immigration: 1^st^ generation immigrant from North Africa | 0.000 | |
| Paternal socioeconomic status | 0.000 | |
| Urbanicity | 0.000 | |
| Pollution | 0.000 | |
| Ethnicity: Black in high ethnic density area | -0.001 | |
| **Abbreviations**: CAARMS, Comprehensive Assessment for At Risk Mental States; GAF, Global Assessment of Functioning; PRS, polygenic risk score for schizophrenia. | | |

**eFigure 5** Calibration plots for Cox proportional hazards models (prognosis). Each calibration plot is derived from all prediction data from test sets across our repeated, nested cross-validation framework. This may lead to narrower CIs than any single calibration plot. **A** Clinical **B** PPS **C** PRS **D** Clinical+PPS **E** Clinical+PRS **F** PPS+PRS **G** Clinical+PPS+PRS

**A**

**B**

**C**

**D**

**E**

**F**

**G**

**eFigure 6** Calibration plots for random survival forest models (prognosis). Each calibration plot is derived from all prediction data from test sets across our repeated, nested cross-validation framework. This may lead to narrower CIs than any single calibration plot. **A** Clinical **B** PPS **C** PRS **D** Clinical+PPS **E** Clinical+PRS **F** PPS+PRS **G** Clinical+PPS+PRS

**A**

**B**

**C**

**D**

**E**

**F**

**G**

| **eTable 10** Sensitivity analysis results | | | | | | | | | | | | | | | | | | | | | | | | |  | | | | | | |
| --- | --- | --- | --- | --- | --- | --- | --- | --- | --- | --- | --- | --- | --- | --- | --- | --- | --- | --- | --- | --- | --- | --- | --- | --- | --- | --- | --- | --- | --- | --- | --- |
| **Model** | **C-index** | **Balanced Accuracy** | | **Sensitivity** | | | | **Specificity** | | | **PPV** | | | **NPV** | | **Calibration**  **Intercept** | | | **Calibration**  **Slope** | | | | **Brier**  **Score** | | | | **ICI** | | |  |  |
| With between-site harmonisation | | | | | | | | | | | | | | | | | | | | | | | | | | | | |  |  |  |
| **PPS** | 0.86  (0.85-0.87) | | 77.7%  (76.4%-79%) | | | 73.6%  (70.8%-76.5%) | | | 81.8%  (80.6%-83.0%) | 44%  (41.2%-46.8%) | | | 94.1%  (93.3%-94.8%) | | | | 1.73  (1.57-1.9) | | | 0.51  (0.46-0.56) | 0.15  (0.14-0.15) | | | | | | 0.14  (0.13-0.15) | |  |  |  |
| **PRS** | 0.67  (0.65-0.69) | | 61.0%  (59.2%-62.7%) | | | 62.5%  (58.9%-66.2%) | | | 59.4%  (58.1%-60.7%) | 22.8%  (21.1%-24.6%) | | | 89.0%  (87.8%-90.2%) | | | | 1.67  (1.58-1.76) | | | 0.99  (0.82-1.16) | 0.23  (0.22-0.23) | | | | | | 0.30  (0.29-0.31) | |  |  |  |
| **PPS+PRS** | 0.87  (0.85-0.88) | | 77.8%  (76.2%-79.3%) | | | 72.4%  (69.4%-75.4%) | | | 83.2%  (81.9%-84.5%) | 45.7%  (42.5%-48.8%) | | | 93.9%  (93.2%-94.7%) | | | | 1.67  (1.49-1.84) | | | 0.53  (0.48-0.58) | 0.14  (0.13-0.15) | | | | | | 0.13  (0.12-0.14) | |  |  |  |
| White European ancestry only | | | | | | | | | | | | | | | | | | | | | | | | | | | | | | | |
| **PPS** | 0.89  (0.88-0.90) | 79.8%  (78.2%-81.5%) | | | 78.6%  (75.5%-81.8%) | | 81.1%  (79.7%-82.4%) | | | | | 44.8%  (41.8%-47.8%) | | | 95.2%  (94.5%-95.9%) | | | 1.46  (1.33-1.59) | | 0.96  (0.84-1.08) | | 0.13  (0.12-0.14) | | | | 0.15  (0.13-0.16) | | |  |  |  |
| **PRS** | 0.62  (0.60-0.64) | 54.7%  (52.5%-56.9%) | | | 53.5%  (49.3%-57.8%) | | 55.8%  (54.1%-57.5%) | | | | | 19.1%  (17.1%-21.1%) | | | 86.2%  (84.9%-87.5%) | | | 1.67  (1.58-1.76) | | 1.57  (0.91-2.22) | | 0.24  (0.24-0.25) | | | | 0.33  (0.32-0.34) | | |  |  |  |
| **PPS+PRS** | 0.89  (0.88-0.90) | 79.8%  (78.1%-81.5%) | | | 77.2%  (74.2%-80.2%) | | 82.5%  (81.1%-83.9%) | | | | | 46.4%  (43.4%-49.5%) | | | 94.9%  (94.1%-95.6%) | | | 1.45  (1.31-1.58) | | 0.96  (0.86-1.06) | | | | 0.13  (0.12-0.13) | | | | 0.14  (0.13-0.15) | | |  |

| **Random forest** | | | | | | | | | | | | | | | | | | | | |  |  |
| --- | --- | --- | --- | --- | --- | --- | --- | --- | --- | --- | --- | --- | --- | --- | --- | --- | --- | --- | --- | --- | --- | --- |
| With between-site harmonisation | | | | | | | | | | | | | | | | | |  | | | |  |
| **PPS** | 0.86  (0.85-0.88) | 70.6%  (68.7%-72.4%) | | 48.6%  (44.9%-52.2%) | | 92.6%  (91.7%-93.4%) | | | 55.7%  (52.3%-59%) | | 90.1%  (88.9%-91.2%) | 0.09  (-0.06-0.25) | 0.67  (0.6-0.75) | 0.11  (0.10-0.11) | | | 0.07  (0.07-0.08) | |  |  |  |  |
| **PRS** | 0.67  (0.65-0.69) | 50.2%  (49.9%-50.5%) | | 0.6%  (0%-1.2%) | | 99.8%  (99.7%-99.9%) | | | N/A | | 83.7%  (82.6%-84.9%) | 0.02  (-0.09-0.13) | 1.08  (0.9-1.26) | 0.13  (0.12-0.14) | | | 0.07  (0.06-0.08) | |  |  |  |  |
| **PPS+PRS** | 0.86  (0.80-0.91) | 60.7%  (57.2%-64.2%) | | 26.8%  (19.2%-34.4%) | | 94.6%  (91.8%-97.4%) | | | 51.8%  (36.5%-67.1%) | | 87%  (85.8%-88.3%) | -0.06  (-0.64-0.52) | 0.92  (0.52-1.33) | 0.11  (0.09-0.13) | | | 0.07  (0.05-0.09) | |  |  |  |  |
| White European ancestry only | | | | | | | | | | | | | | | | | | | | | | |
| **PPS** | 0.88  (0.85-0.91) | | 74.5%  (70.6%-78.4%) | | 52.1%  (45.8%-58.4%) | | 96.8%  (94.6%-99.1%) | 77.8%  (63.1%-92.6%) | | 91.3%  (90%-92.5%) | | -0.03  (-0.41-0.34) | 0.74  (0.58-0.90) | | 0.09  (0.07-0.1) | 0.06  (0.05-0.08) | | | |  |  |  |
| **PRS** | 0.57  (0.51-0.62) | | 50.9%  (48.3%-53.6%) | | 4.1%  (-1.2%-9.4%) | | 97.8%  (97.1%-98.5%) | 16.7%  (-4.0%-37.3%) | | 84%  (81.7%-86.2%) | | 1.35  (0.99-1.71) | 0.09  (-0.01-0.19) | | 0.16  (0.15-0.17) | 0.16  (0.14-0.18) | | | |  |  |  |
| **PPS+PRS** | 0.89  (0.86-0.92) | | 73.1%  (69.5%-76.7%) | | 50.1%  (44.3%-56.0%) | | 96.1%  (93.2%-99.0%) | 74.7%  (59.0%-90.5%) | | 90.8%  (89.1%-92.5%) | | -0.08  (-0.47-0.32) | 0.84  (0.67-1.02) | | 0.09  (0.07-0.10) | 0.07  (0.05-0.09) | | | |  |  |  |

| **Model** | **C-index** | **Calibration Intercept** | **Calibration Slope** | **Brier Score** | **ICI** |
| --- | --- | --- | --- | --- | --- |
| **Cox proportional hazards model** |  |  |  |  |  |
| With between-site harmonisation |  |  |  |  |  |
| **Clinical** | 0.54  (0.53-0.55) | -65.97  (-143.46-11.51) | 73.04  (-12.54-158.63) | 0.52  (0.48-0.55) | 0.54  (0.53-0.55) |
| **PPS** | 0.55  (0.53-0.57) | -48.9  (-116.59-18.79) | 77.91  (-11.73-167.54) | 0.52  (0.49-0.55) | 0.55  (0.53-0.57) |
| **PRS** | 0.57  (0.55-0.58) | -1.59  (-4.78-1.61) | -0.23  (-3.33-2.86) | 0.52  (0.49-0.55) | 0.57  (0.55-0.58) |
| **Clinical+PPS** | 0.55  (0.53-0.57) | -79.09  (-263.93-105.76) | 63.25  (-84.49-211.00) | 0.52  (0.49-0.56) | 0.55  (0.53-0.57) |
| **Clinical+PRS** | 0.55  (0.53-0.57) | -31.56  (-77.96-14.85) | 26.41  (-11.33-64.14) | 0.52  (0.48-0.55) | 0.55  (0.53-0.57) |
| **PPS+PRS** | 0.55  (0.53-0.57) | 89.46  (-48.49-227.40) | -127.26  (-268.41-13.89) | 0.52  (0.49-0.55) | 0.55  (0.53-0.57) |
| **All** | 0.55  (0.53-0.57) | -23534.66  (-69566.45-22497.12) | 16319.05  (-12902.44-45540.55) | 0.52  (0.49-0.56) | 0.55  (0.53-0.57) |
| White European ancestry only |  |  |  |  |  |
| **Clinical** | 0.61  (0.59-0.63) | -17.05  (-27.29--6.81) | 16.43  (6.03-26.83) | 0.52  (0.49-0.56) | 0.39  (0.32-0.46) |
| **PPS** | 0.58  (0.56-0.59) | -77.83  (-150.63--5.03) | 79.85  (5.97-153.74) | 0.53  (0.49-0.56) | 0.65  (0.52-0.78) |
| **PRS** | 0.58  (0.56-0.59) | -3.63  (-5.2--2.05) | 1.61  (0.22-3) | 0.52  (0.49-0.56) | 0.84  (0.83-0.86) |
| **Clinical+PPS** | 0.58  (0.56-0.60) | -49.68  (-140.31-40.95) | 55.81  (-47.41-159.03) | 0.52  (0.49-0.55) | 0.45  (0.34-0.56) |
| **Clinical+PRS** | 0.57  (0.55-0.59) | -18.96  (-35.76--2.16) | 16.56  (2.12-31.00) | 0.52  (0.49-0.55) | 0.42  (0.35-0.48) |
| **PPS+PRS** | 0.58  (0.56-0.60) | -6.67  (-13.35-0) | 6.77  (-1.91-15.45) | 0.52  (0.49-0.55) | 0.63  (0.53-0.73) |
| **All** | 0.58  (0.56-0.60) | -1.65  (-20.34-17.04) | 1.50  (-19.09-22.08) | 0.52  (0.49-0.55) | 0.48  (0.37-0.59) |
| **Random survival forest** |  |  |  |  |  |
| With between-site harmonisation |  |  |  |  |  |
| **Clinical** | 0.59  (0.57-0.61) | -0.33  (-0.45--0.2) | 0.55  (0.34-0.76) | 0.15  (0.14-0.16) | 0.1  (0.09-0.11) |
| **PPS** | 0.58  (0.57-0.59) | -0.34  (-0.46--0.22) | -0.21  (-0.34--0.08) | 0.16  (0.16-0.17) | 0.12  (0.11-0.13) |
| **PRS** | 0.59  (0.57-0.60) | -0.34  (-0.47--0.21) | 0.30  (0.17-0.42) | 0.16  (0.15-0.17) | 0.12  (0.11-0.13) |
| **Clinical+PPS** | 0.56  (0.55-0.58) | -0.33  (-0.45--0.22) | -0.29  (-0.73-0.15) | 0.15  (0.14-0.16) | 0.1  (0.09-0.11) |
| **Clinical+PRS** | 0.58  (0.57-0.60) | -0.41  (-0.54--0.28) | 0.4  (0.27-0.53) | 0.16  (0.15-0.16) | 0.12  (0.11-0.13) |
| **PPS+PRS** | 0.57  (0.56-0.58) | -0.36  (-0.48--0.24) | -0.1  (-0.24-0.05) | 0.16  (0.15-0.17) | 0.12  (0.11-0.13) |
| **All** | 0.57  (0.56-0.58) | -0.38  (-0.5--0.25) | 0.36  (0.20-0.52) | 0.15  (0.15-0.16) | 0.1  (0.09-0.12) |
| White European ancestry only |  |  |  |  |  |
| **Clinical** | 0.57  (0.55-0.58) | -1.67  (-1.78--1.55) | 0.12  (-0.02-0.26) | 0.26  (0.26-0.27) | 0.32  (0.30-0.33) |
| **PPS** | 0.56  (0.55-0.58) | -0.33  (-0.45--0.21) | 0.09  (-0.03-0.21) | 0.16  (0.15-0.17) | 0.11  (0.10-0.12) |
| **PRS** | 0.59  (0.57-0.60) | -0.34  (-0.47--0.21) | 0.29  (0.17-0.41) | 0.16  (0.15-0.17) | 0.12  (0.11-0.13) |
| **Clinical+PPS** | 0.58  (0.56-0.60) | -0.35  (-0.47--0.23) | 0.33  (0.17-0.49) | 0.15  (0.15-0.16) | 0.1  (0.09-0.11) |
| **Clinical+PRS** | 0.58  (0.56-0.60) | -0.41  (-0.54--0.28) | 0.40  (0.26-0.53) | 0.16  (0.15-0.16) | 0.12  (0.11-0.13) |
| **PPS+PRS** | 0.58  (0.56-0.60) | -0.33  (-0.45--0.21) | 0.01  (-0.12-0.14) | 0.16  (0.15-0.16) | 0.11  (0.10-0.13) |
| **All** | 0.58  (0.56-0.60) | -0.37  (-0.49--0.24) | 0.39  (0.19-0.58) | 0.15  (0.15-0.16) | 0.11  (0.10-0.12) |

| **eTable 11** Model performance for prognosis models of remission from the CHR-P state. | | | | | | | | | | | |
| --- | --- | --- | --- | --- | --- | --- | --- | --- | --- | --- | --- |
| **Model** | **C-index** | **Balanced accuracy** | **Sensitivity** | **Specificity** | **PPV** | **NPV** | **Calibration intercept** | **Calibration slope** | **Brier score** | **ICI** |  |
| **Logistic regression** | | | | | | | | | | |  |
| **Clinical** | 0.53  (0.52-0.54) | 47.6%  (46.6%-48.7%) | 49.1%  (39.9%-58.3%) | 46.2%  (36.9%-55.6%) | 51.9%  (50.4%-53.4%) | 42.2%  (40.1%-44.3%) | -0.17  (-0.23--0.12) | -1.53  (-2.35--0.71) | 0.25  (0.25-0.25) | 0.12  (0.11-0.13) |  |
| **PPS** | 0.55  (0.53-0.56) | 52.5%  (51%-53.9%) | 58.8%  (54.9%-62.8%) | 46.1%  (42%-50.2%) | 56.4%  (54.5%-58.3%) | 48.9%  (46.8%-51%) | -0.17  (-0.23--0.11) | 0.5  (0.13-0.87) | 0.25  (0.25-0.26) | 0.11  (0.1-0.12) |  |
| **PRS** | 0.57  (0.55-0.59) | 48.3%  (46.3%-50.4%) | 49.8%  (47.4%-52.2%) | 46.9%  (43.9%-49.9%) | 52.7%  (50.3%-55.1%) | 43.9%  (41.4%-46.4%) | -0.17  (-0.22--0.11) | -0.75  (-7.58-6.08) | 0.25  (0.25-0.26) | 0.12  (0.11-0.13) |  |
| **Clinical+PPS** | 0.55  (0.54-0.57) | 52.3%  (50.6%-53.9%) | 60.9%  (57.2%-64.6%) | 43.6%  (39.3%-47.9%) | 56.3%  (54.4%-58.3%) | 48.7%  (46.3%-51%) | -0.17  (-0.23--0.12) | 0.63  (0.16-1.11) | 0.25  (0.25-0.26) | 0.11  (0.1-0.12) |  |
| **Clinical+PRS** | 0.55  (0.54-0.57) | 52.3%  (50.6%-53.9%) | 60.9%  (57.2%-64.6%) | 43.6%  (39.3%-47.9%) | 56.3%  (54.4%-58.3%) | 48.7%  (46.3%-51%) | -0.17  (-0.23--0.12) | 0.63  (0.16-1.11) | 0.25  (0.25-0.26) | 0.11  (0.1-0.12) |  |
| **PPS+PRS** | 0.55  (0.54-0.56) | 51.9%  (50.4%-53.4%) | 56.8%  (52.6%-61%) | 46.9%  (42.4%-51.5%) | 56.2%  (54.2%-58.1%) | 48%  (45.9%-50.1%) | -0.17  (-0.22--0.12) | 0.31  (-0.45-1.07) | 0.25  (0.25-0.26) | 0.11  (0.1-0.12) |  |
| **All** | 0.55  (0.54-0.57) | 52.3%  (50.6%-53.9%) | 60.9%  (57.2%-64.6%) | 43.6%  (39.3%-47.9%) | 56.3%  (54.4%-58.3%) | 48.7%  (46.3%-51%) | -0.17  (-0.23--0.12) | 0.63  (0.16-1.11) | 0.25  (0.25-0.26) | 0.11  (0.1-0.12) |  |
| **Random forest** | | | | | | | | | | |  |
| **Clinical** | 0.53  (0.51-0.54) | 50.8%  (48.1%-53.5%) | 53.5%  (49.7%-57.4%) | 48%  (39.4%-56.7%) | 57.8%  (52.6%-63%) | 43.6%  (39.3%-47.9%) | -0.27  (-0.42--0.11) | -0.02  (-0.13-0.09) | 0.3  (0.28-0.31) | 0.2  (0.18-0.22) |  |
| **PPS** | 0.58  (0.55-0.61) | 53.4%  (48.2%-58.5%) | 55%  (47.7%-62.3%) | 51.8%  (41.5%-62%) | 60.3%  (54.1%-66.5%) | 46.6%  (39.2%-54%) | -0.29  (-0.48--0.1) | 0.08  (-0.16-0.33) | 0.28  (0.26-0.31) | 0.19  (0.15-0.24) |  |
| **PRS** | 0.54  (0.51-0.56) | 48.4%  (44%-52.8%) | 52.5%  (46.4%-58.5%) | 44.3%  (34.5%-54.1%) | 55.6%  (49.5%-61.7%) | 41.1%  (35.3%-47%) | -0.26  (-0.44--0.08) | 0  (-0.07-0.08) | 0.32  (0.3-0.35) | 0.24  (0.2-0.28) |  |
| **Clinical+PPS** | 0.58  (0.56-0.61) | 52.4%  (48.4%-56.3%) | 58.1%  (53.6%-62.6%) | 46.6%  (39.6%-53.7%) | 59%  (55.2%-62.9%) | 45.7%  (38.4%-53%) | -0.27  (-0.45--0.1) | 0.21  (-0.24-0.67) | 0.27  (0.24-0.29) | 0.13  (0.1-0.16) |  |
| **Clinical+PRS** | 0.56  (0.54-0.59) | 45.4%  (42.2%-48.6%) | 49.3%  (43.9%-54.6%) | 41.5%  (36.9%-46.1%) | 52.5%  (46.5%-58.4%) | 38.4%  (34.5%-42.4%) | -0.29  (-0.45--0.13) | -0.19  (-0.36--0.02) | 0.29  (0.28-0.31) | 0.2  (0.16-0.24) |  |
| **PPS+PRS** | 0.57  (0.52-0.62) | 49.3%  (43.2%-55.3%) | 55.7%  (50.5%-60.8%) | 42.8%  (34%-51.7%) | 56.2%  (48.7%-63.8%) | 42.1%  (35.5%-48.7%) | -0.29  (-0.46--0.13) | -0.02  (-0.4-0.35) | 0.28  (0.26-0.3) | 0.18  (0.13-0.22) |  |
| **All** | 0.56  (0.53-0.59) | 47.4%  (42.6%-52.3%) | 55.8%  (50.7%-60.9%) | 39.1%  (32.1%-46%) | 54.7%  (50.7%-58.7%) | 40.3%  (32.1%-48.5%) | -0.29  (-0.45--0.14) | -0.06  (-0.42-0.3) | 0.27  (0.26-0.29) | 0.17  (0.13-0.21) |  |

**eResults 1** Post hoc sample size calculation using pmsampsize

**Detection**

**PPS**: pmsampsize(type = "b", cstatistic = 0.88, parameters = 17, prevalence = 344/411); n=488

**PRS**: pmsampsize(type = "b", cstatistic = 0.62, parameters = 1, prevalence = 344/411); n=355

**PPS+PRS**: pmsampsize(type = "b", cstatistic = 0.88, parameters = 19, prevalence = 344/411); n=546

**Prognosis**

**Clinical:** pmsampsize(type="s",

nagrsquared=0.072,

parameters = 1,

rate=0.19*3,

shrinkage=0.9,

timepoint = 3,

meanfup=644/365); n=141

**PPS**: pmsampsize(type="s",

nagrsquared =0.23,

parameters = 17,

rate=0.19*3,

shrinkage=0.9,

timepoint = 3,

meanfup=644/365); n=681

**PRS**: pmsampsize(type="s",

nagrsquared =0.023,

parameters = 1,

rate=0.19*3,

shrinkage=0.9,

timepoint = 3,

meanfup=644/365); n=445

**Clinical+PPS:** pmsampsize(type="s",

nagrsquared =0.31,

parameters = 21,

rate=0.19*3,

shrinkage=0.9,

timepoint = 3,

meanfup=644/365); n=595

**Clinical+PRS:** pmsampsize(type="s",

nagrsquared =0.097,

parameters = 2,

rate=0.19*3,

shrinkage=0.9,

timepoint = 3,

meanfup=644/365); n=205

**PPS+PRS**: pmsampsize(type="s",

nagrsquared =0.26,

parameters = 22,

rate=0.19*3,

shrinkage=0.9,

timepoint = 3,

meanfup=644/365); n=765

**Clinical+PPS+PRS:** pmsampsize(type="s",

nagrsquared =0.34,

parameters = 15,

rate=0.19*3,

shrinkage=0.9,

timepoint = 3,

meanfup=644/365); n=380

**eReferences**

1.	Yung AR, Yuen HP, McGorry PD, et al. Mapping the onset of psychosis: the Comprehensive Assessment of At-Risk Mental States. *The Australian and New Zealand Journal of Psychiatry*. 2005;39(11-12):964-971. doi:10.1111/j.1440-1614.2005.01714.x

2.	First MB, Gibbon M. The Structured Clinical Interview for DSM-IV Axis I Disorders (SCID-I) and the Structured Clinical Interview for DSM-IV Axis II Disorders (SCID-II). *Comprehensive handbook of psychological assessment, Vol 2: Personality assessment*. Published online 2004:134-143.

3.	Wechsler D. *Wechsler Abbreviated Scale of Intelligence, Second Edition (WASI-II)*. NCS Pearson; 2011.

4.	Radua J, Ramella-Cravaro V, Ioannidis JPA, et al. What causes psychosis? An umbrella review of risk and protective factors. *World psychiatry : official journal of the World Psychiatric Association (WPA)*. 2018;17(1):49-66. doi:10.1002/wps.20490

5.	Eurostat (European Commission). *Labour Force Survey in the EU, Candidate and EFTA Countries — Main Characteristics of National Surveys, 2014*. Eurostat; 2015. Accessed March 22, 2019. https://ec.europa.eu/eurostat/documents/7870049/9443652/KS-FT-18-009-EN-N.pdf/964ae130-aa7e-4a5d-a221-d14fd62bd511

6.	Oliver D, Radua J, Reichenberg A, Uher R, Fusar-Poli P. Psychosis Polyrisk Score (PPS) for the Detection of Individuals At-Risk and the Prediction of Their Outcomes. *Frontiers in psychiatry*. 2019;10:174. doi:10.3389/fpsyt.2019.00174

7.	Oliver D, Spada G, Englund A, et al. Real-world digital implementation of the Psychosis Polyrisk Score (PPS): A pilot feasibility study. *Schizophrenia Research*. 2020;226:176-183. doi:10.1016/j.schres.2020.04.015

8.	Zheng Z, Liu S, Sidorenko J, et al. Leveraging functional genomic annotations and genome coverage to improve polygenic prediction of complex traits within and between ancestries. *Nat Genet*. 2024;56(5):767-777. doi:10.1038/s41588-024-01704-y

9.	Trubetskoy V, Pardiñas AF, Qi T, et al. Mapping genomic loci implicates genes and synaptic biology in schizophrenia. *Nature*. 2022;604(7906):502-508. doi:10.1038/s41586-022-04434-5

10.	Vickers AJ, Van Calster B, Steyerberg EW. Net benefit approaches to the evaluation of prediction models, molecular markers, and diagnostic tests. *BMJ (Clinical Research Ed)*. 2016;352:i6. doi:10.1136/bmj.i6

11.	Vickers AJ, van Calster B, Steyerberg EW. A simple, step-by-step guide to interpreting decision curve analysis. *Diagn Progn Res*. 2019;3(1):18. doi:10.1186/s41512-019-0064-7
